# Mechanical Properties and Microstructure of Bonded Joints and Hybrid Structures with a 3D-Printed Honeycomb Core Modified with an Epoxy Matrix Filled with Recycled Polyurethane Foam

**DOI:** 10.3390/ma19183935

**Published:** 2026-09-16

**Authors:** Michal Penc, Miroslav Müller, Jiří Marčan, Rajesh Kumar Mishra, Jaroslava Svobodová, Petr Jirků, Anna Rudawska, Petr Valášek

**Affiliations:** 1 Faculty of Engineering, Czech University of Life Sciences, Kamýcká 129, 165 00 Prague, Czech Republic; penc@tf.czu.cz (M.P.); marcan@tf.czu.cz (J.M.); svobodovajaroslava@tf.czu.cz (J.S.); jirkup@tf.czu.cz (P.J.); valasekp@tf.czu.cz (P.V.); 2 Faculty of Mechanical Engineering, J. E. Purkyne Universty in Usti nad Labem, Pasteurova 3334/7, 400 96 Usti nad Labem, Czech Republic; 3 Faculty of Mechanical Engineering, Lublin University of Technology, Nadbystrzycka 36, 20-618 Lublin, Poland; a.rudawska@pollub.pl

**Keywords:** mechanical recycling, polyurethane foam, 3D printing, honeycomb core, epoxy composite, adhesive bond strength, scanning electron microscopy (SEM), bonded joints

## Abstract

This article examines the reuse of waste polyurethane foam (PUF) as a material in line with circular economy principles. The main objective was to evaluate how crushed polyurethane filler of different bulk densities—35 kg·m^−3^ (PUF35), 60 kg·m^−3^ (PUF60), and their blends at 1–5 wt%—affects the mechanical behaviour and structural integrity of hybrid composite systems and bonded laminated joints. An epoxy resin matrix was combined with 3D-printed polylactide (PLA) honeycomb structures, with rectangular and hexagonal core geometries. Static tensile tests showed that the blended filler (PUF35/60) preserves tensile strength and increases the modulus of elasticity for both core geometries, reaching maximum values at 4 wt% (3.8 GPa for rectangular, 3.6 GPa for hexagonal cores). In bonded lap joints, 1 wt% PUF35 resulted in the highest tensile adhesive bond strength (13.3 MPa). SEM confirmed a high-quality phase interface and continuous adhesive contact between the epoxy matrix and the 3D-printed PLA surface, with dominant cohesive failure and effective mechanical anchoring of foam particles, even near local printing defects. The results confirm mechanically recycled PUF as a promising filler for advanced sandwich structures and adhesive systems.

## 1. Introduction

Polyurethane foams (PUF) are widely used polymer materials due to their low density, high energy absorption, favourable mechanical properties, and thermal insulation. They include soft and rigid foams and thermoplastic polyurethanes (TPU), with applications in furniture, construction, automotive and aerospace industries, healthcare, footwear, and adhesives [1,2,3,4]. PUFs are highly cross-linked polymers based on isocyanates and multifunctional polyols and often contain additives, such as flame retardants, which complicate their recycling and reuse [5,6].

Increasing polyurethane consumption has resulted in growing amounts of waste and associated environmental concerns [7,8,9,10]. Global polyurethane production increased from approximately 18 million metric tons in 2016 to 26 million metric tons in 2022 and is expected to reach approximately 31 million metric tons annually by 2030 [6,11]. Although recycled PUF is used, for example, in carpet underlay [7], substantial amounts are still landfilled or incinerated [12,13,14]. These methods are environmentally problematic because PUF biodegrades slowly and its incineration can produce hazardous substances, including hydrogen cyanide and nitrogen oxides [15,16], highlighting the need for effective recycling.

PUF recycling involves chemical and mechanical methods [17]. Chemical recycling includes acidolysis [2,15], glycolysis [18,19], hydrolysis [20], and aminolysis [21], which break down the polymer network into low-molecular-weight products. However, high energy requirements and complex product purification limit their wider industrial application [17,22]. Mechanical recycling provides a simpler and more economical alternative, converting PUF into particles suitable as fillers or raw materials for new composites [17,23,24]. Common methods include rebonding, compression moulding, and injection moulding [25,26], while mechanical grinding represents a promising approach for material recovery due to the low density and high volume of PUF waste and the limitations of chemical recycling [27,28,29].

Mechanically recycled PUF has been investigated as a filler in adhesives, polymer matrices, and composite systems [30,31,32]. Hýsek et al. [4] incorporated recycled rigid PUR/PIR foam into one-component polyurethane adhesives for wood bonding, improving heat resistance after boiling while reducing shear strength after cold-water conditioning [4]. Mansouri and Pizzi [30] used micro-ground recycled flexible PUF in UF and PF adhesives, increasing bond strength and water resistance [30]. In composite systems, Seo et al. [32] used mechanically ground PUF as an interlayer filler in recycled carbon-fibre/epoxy composites, improving mechanical interlocking and interlaminar adhesion and consequently several mechanical properties [32]. Liang et al. [33] produced sandwich composites from recycled rigid PUF and waste polyester-cotton fabric, obtaining favourable mechanical, thermal, acoustic, fire-resistance, and water-repellency properties [33]. Selvaraj et al. [12] incorporated recycled rigid PUF into a bioepoxy matrix with vermiculite, improving impact toughness, thermal stability, electromagnetic shielding, and acoustic properties while reducing thermal conductivity and flammability [12].

Overall, these studies demonstrate the potential of mechanically recycled PUF as a filler in polymer adhesives and composites, providing mechanical and functional benefits while supporting polyurethane waste reuse and circular-economy principles [29,30,31,32].

Although mechanically recycled PUF has been successfully used in adhesives, polymer matrices, and composite materials, its application as a filler in hybrid composite systems has been studied only to a limited extent so far. At the same time, there is a lack of a comprehensive evaluation of the effect of various fractions of mechanically recycled PUF on the mechanical properties of such systems.

The 3D-printed honeycomb core was incorporated into the hybrid composite system to provide structural stiffness to the epoxy matrix modified with recycled PUF, as honeycomb structures are characterized by high specific stiffness and efficient load transfer [34,35]. The mechanical behaviour of honeycomb-based structures is influenced not only by the core geometry but also by the interaction between the honeycomb cell walls and the surrounding or adjoining polymeric phase. Kendall et al. [36] demonstrated that adhesive bonding can constrain cell-wall deformation and facilitate load transfer between structural components, thereby affecting the stiffness and overall mechanical response of honeycomb structures. In honeycomb-based structures, the mechanical performance is influenced not only by the geometry and properties of the core but also by the quality of the bond formed at the honeycomb interface. Previous studies have demonstrated that the morphology and integrity of the adhesive bond line, including adhesive fillet formation, interfacial contact, and the presence of voids, can significantly affect the mechanical performance of honeycomb structures [37,38,39].

PLA was selected as the core material due to its wide availability, low cost, good FDM processability, and suitable mechanical properties. Two core geometries were investigated: hexagonal honeycomb and rectangular (grid). The hexagonal geometry provides a more uniform stress distribution, whereas the rectangular geometry can provide higher strength and stiffness in the loading direction [40,41,42,43]. Both geometries were used to evaluate the effect of core geometry on the mechanical behaviour of hybrid composite systems modified with recycled PUF.

The main objective of this study was therefore to investigate the sustainable utilization of mechanically recycled PUF as a filler in an epoxy matrix and to evaluate its effect in two applications: hybrid composite structures with 3D-printed PLA honeycomb cores and bonded lap joints.

## 2. Materials and Methods

A two-component LH 145 epoxy resin combined with MGS 285 hardener (Havel Composites s.r.o., Svésedlice, Czech Republic) was used as the matrix for the hybrid composite system. The epoxy system is primarily intended for producing high-stress composite parts, particularly in the aerospace industry.

### 2.1. Experimental Procedure

**Figure 1:**
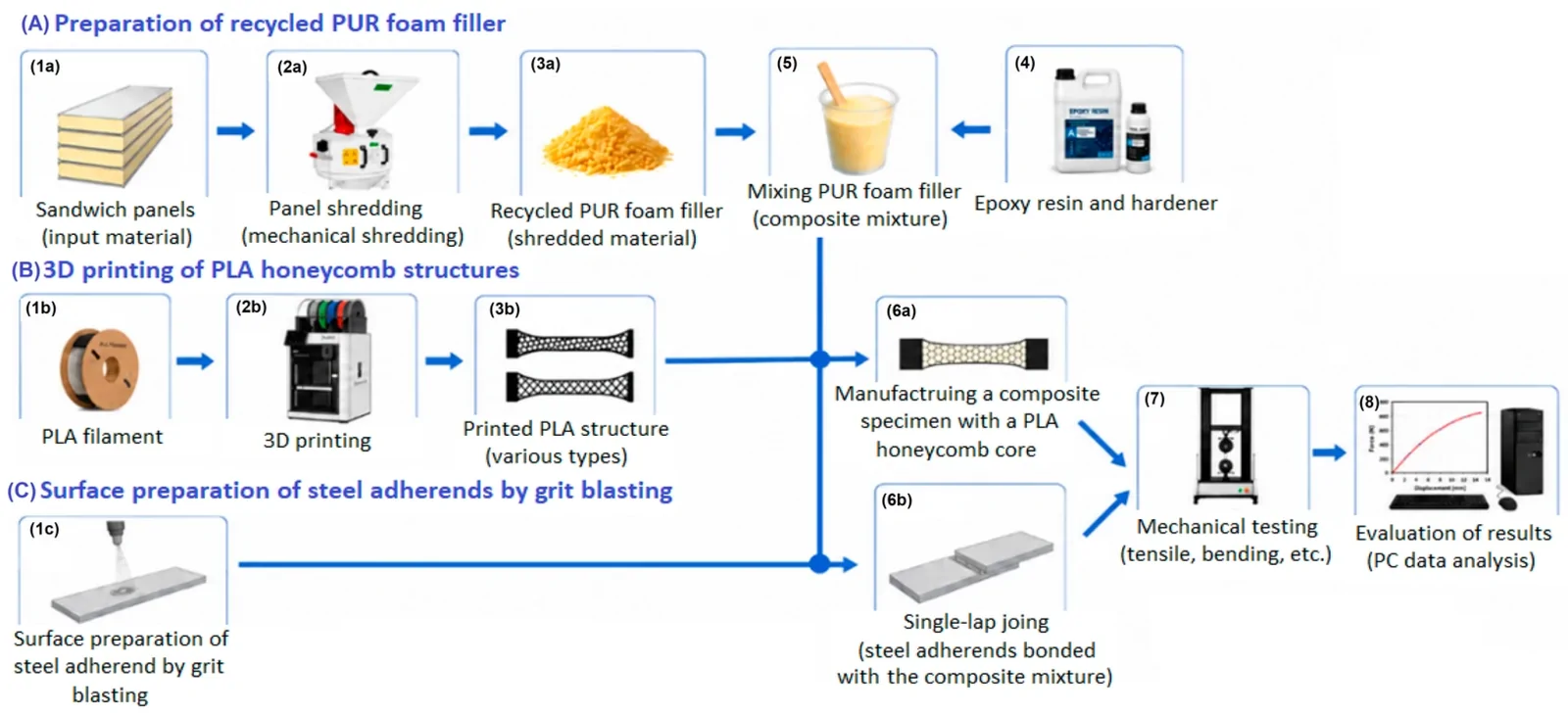
Schematic overview of the experimental procedure adopted in this study.

The experimental procedure of the study is schematically illustrated in Figure 1.

Figure 1A (1–3) describes the preparation of recycled polyurethane foam filler, including the procurement of the feedstock, its mechanical crushing, and the preparation of the particulate filler. Figure 1B (1–3) describes the fabrication of honeycomb structures from PLA using 3D printing, which were subsequently used as reinforcement for hybrid composite specimens. Figure 1C (1) illustrates the surface preparation of steel adherends by shot blasting before fabrication of lap joints. In subsequent steps, a composite mixture was prepared by combining epoxy resin with recycled PUF filler (4–5), from which composite specimens with a honeycomb core (6a) and laminated-bonded joints (6b) were fabricated. The prepared specimens were subjected to mechanical testing (7), and the results were subsequently analysed statistically (8).

### 2.2. Preparation of the Filler

Recycled PUF obtained from sandwich panels supplied by PSP Izoterm s.r.o, (Vysoké Mýto, Czech Republic)., which are commonly used as insulation material in refrigerated truck bodies, was used for preparing particulate fillers. For the experiment, PUF with bulk densities of PUF35 and PUF60, as well as a mixture of PUF35 and PUF60 (PUF35/60), were selected. The recycled material consisted of rigid closed-cell polyurethane foam with densities of 35 kg·m^−3^ (PUF35) and 60 kg·m^−3^ (PUF60). The inclusion of PUF35/60 reflects a realistic scenario for the material recycling of waste sandwich panels, since separating individual PUF types is both time-consuming and costly from an industrial standpoint.

Before recycling, the fibreglass-reinforced plastic outer layer was removed from the sandwich panels to isolate the PUF. The foam was then cleaned of impurities and mechanically crushed in a VM3 vertical hammer mill (TAURUS s.r.o., Chrudim, Czech Republic) equipped with a 200 μm screen. After grinding, the resulting particulate filler was dried and stored in airtight containers until its subsequent use in the preparation of composite mixtures.

### 2.3. Preparation of Printed Universal Test Specimens

Universal test specimens with hexagonal (H) and rectangular grid (GR) cores were manufactured from black AlzaMent PLA filament (Alza.cz a.s., Prague, Czech Republic) using FDM on a Bambu Lab X1 Carbon Combo 3D printer (Shenzhen Tuozhu Technology Co., Ltd., Shenzhen, China). PLA was selected for its good FDM processability and suitability for lightweight structures with a high strength-to-weight ratio.

Honeycomb structures combine low weight, high specific stiffness, favourable strength-to-weight ratio, and good energy absorption, making them suitable for aerospace, automotive, and construction applications [34,35]. Their mechanical properties depend on morphological parameters, including topology, cell size, and relative density, as well as printing parameters such as orientation and fill density, which influence stress distribution, load transfer, and deformation mechanisms [44,45,46,47,48,49,50,51].

Two core geometries were compared: hexagonal and rectangular. The hexagonal geometry provides more uniform stress distribution and lower sensitivity to printing orientation, whereas the rectangular geometry offers higher strength and stiffness in the loading direction but greater anisotropy [40,41,42,43]. Both were used to evaluate the effect of core geometry on hybrid composite systems modified with recycled PUF. Samples were labelled as Sample Type_Filler Weight %_Filler Density, where H and GR denote the hexagonal and rectangular (grid) structures, respectively.

**Figure 2:**
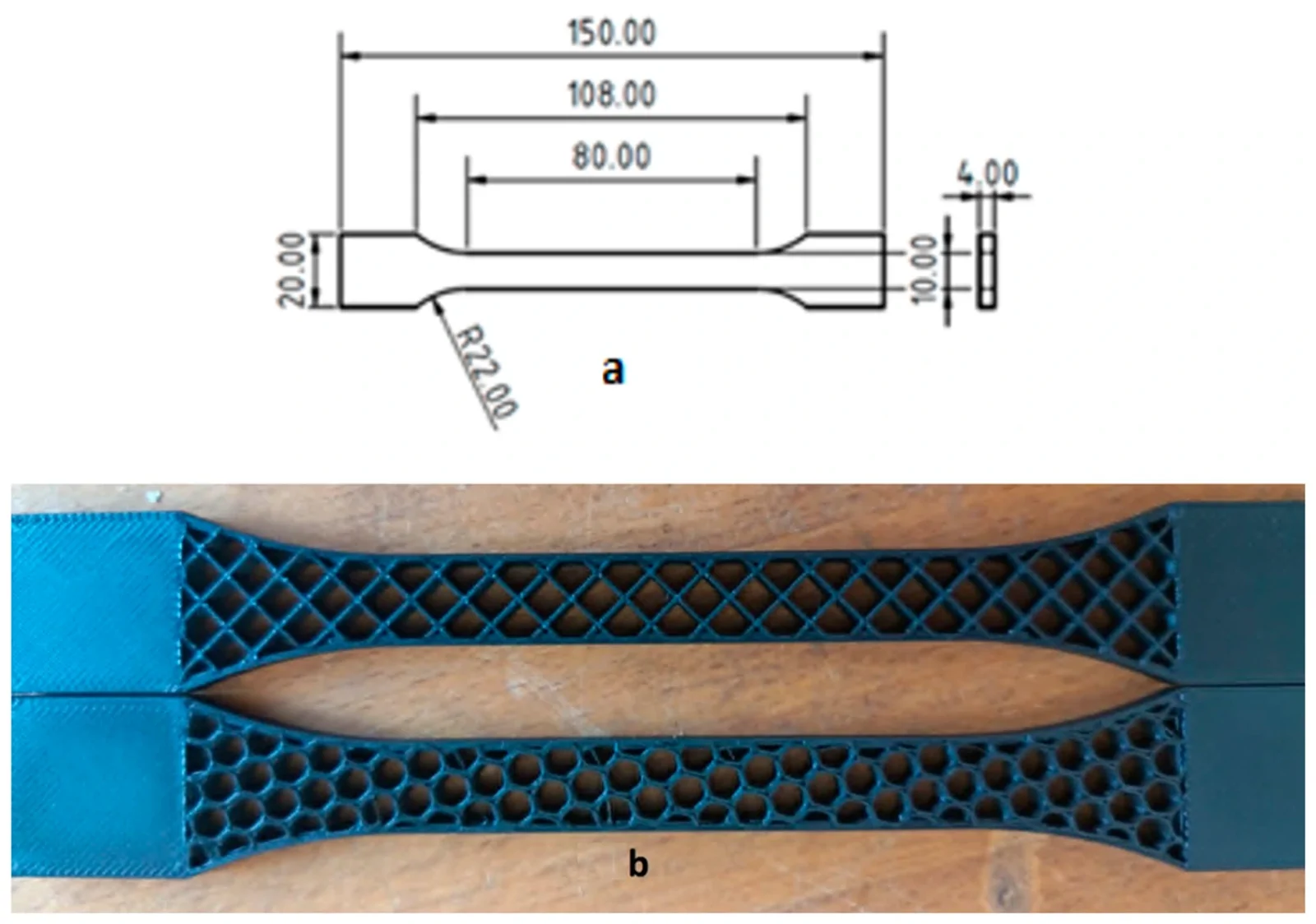
Test specimen dimensions (**a**) and experimental specimens (**b**): rectangular core (**top**), honeycomb core (**bottom**).

The specimens were manufactured by fused deposition molding, involving sequential deposition of molten polymer filament [52,53], using a 0.4 mm nozzle and a fill density of 20%. Their geometry and dimensions complied with ČSN EN ISO 527-2v [54]. Figure 2a shows the specimen dimensions, while Figure 2b presents printed specimens with both core geometries.

### 2.4. Preparation of Samples for Overlapped Joints

**Figure 3:**
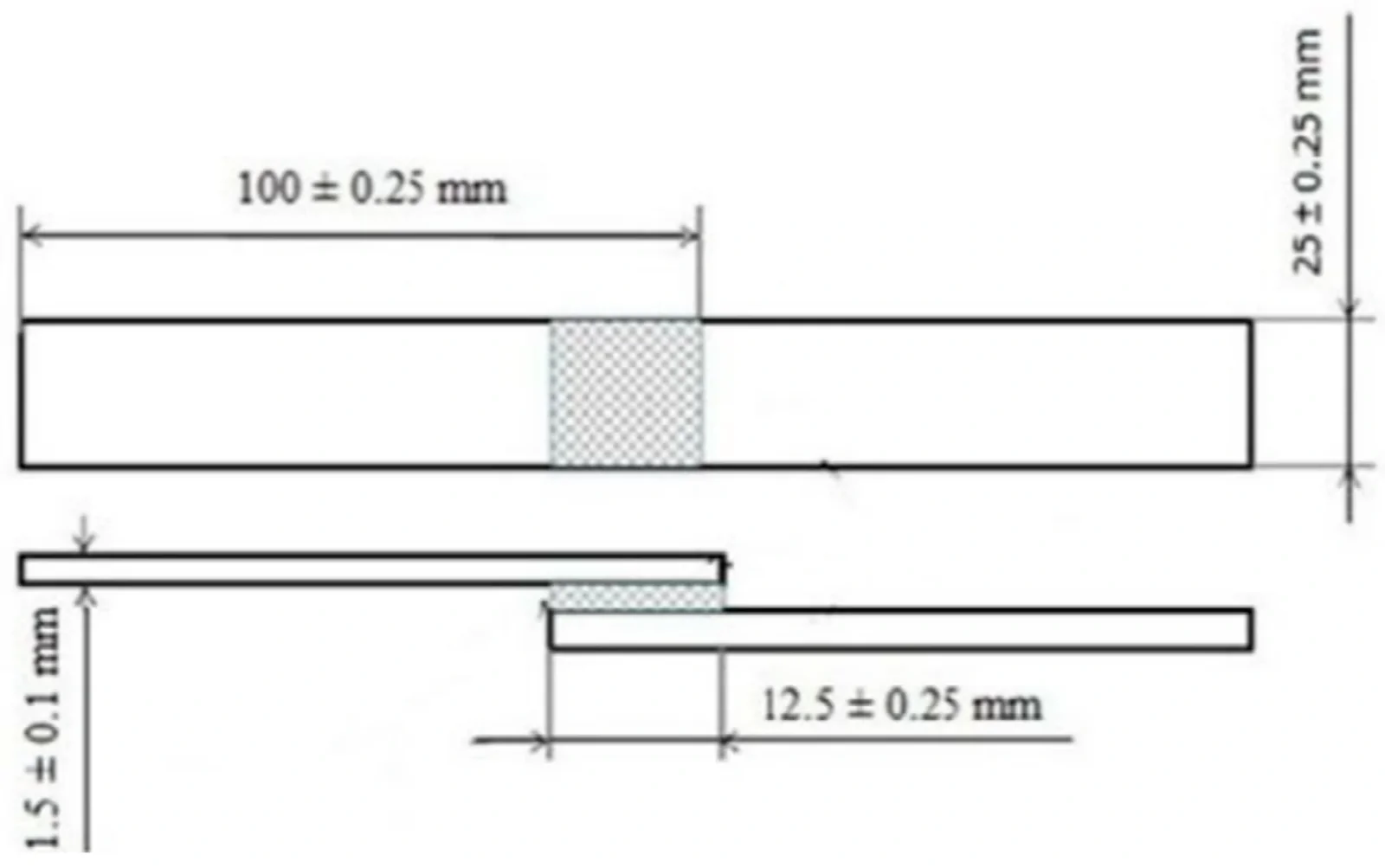
Dimensions of the single-lap joint specimen according to EN 1465 [58].

Overlapped joints (designated by the abbreviation OJ) were prepared from S235J0 structural steel pieces measuring 100 × 25 × 1.5 mm, cut on automatic guillotine shears (Presstechnik s.r.o., Pasov, Czech Republic) in the prototype laboratory. The overlap length was 12.5 ± 0.25 mm in accordance with the ČSN EN 1465 standard [55], as shown in Figure 3. Before adhesive application, the surfaces of the adherends were mechanically treated by abrasive blasting in a closed chamber using natural garnet abrasive with a grit size of 80, resulting in an average surface roughness of Ra = 1.31 ± 0.16 µm. Subsequently, the surfaces were chemically degreased in an acetone bath. This surface treatment is sufficient for adhesive joint applications [56,57]. For unambiguous identification, the samples were labelled in a uniform format: Sample Type_Mass % of Filler_Filler Density.

### 2.5. Preparation of the Composite Mixture and the Application of the Mixture

The composite mixture was prepared by mixing epoxy resin with recycled PUF at a mass concentration of 1–5%. At the same time, reference samples were prepared using epoxy resin without PUF filler. The mass fraction was chosen because determining the volume fraction of the PUF35/60 filler is difficult. Higher filler concentrations were not considered suitable for the intended adhesive and composite applications because the addition of large amounts of particulate PUF would substantially increase the viscosity and reduce the workability of the mixture, while limiting the continuous epoxy matrix required for effective bonding and load transfer. The mixture was homogenised by continuous stirring. Due to the increasing viscosity at higher filler concentrations by weight, vacuum degassing was not performed to ensure the preparation method corresponded to standard industrial production conditions.

**Figure 4:**
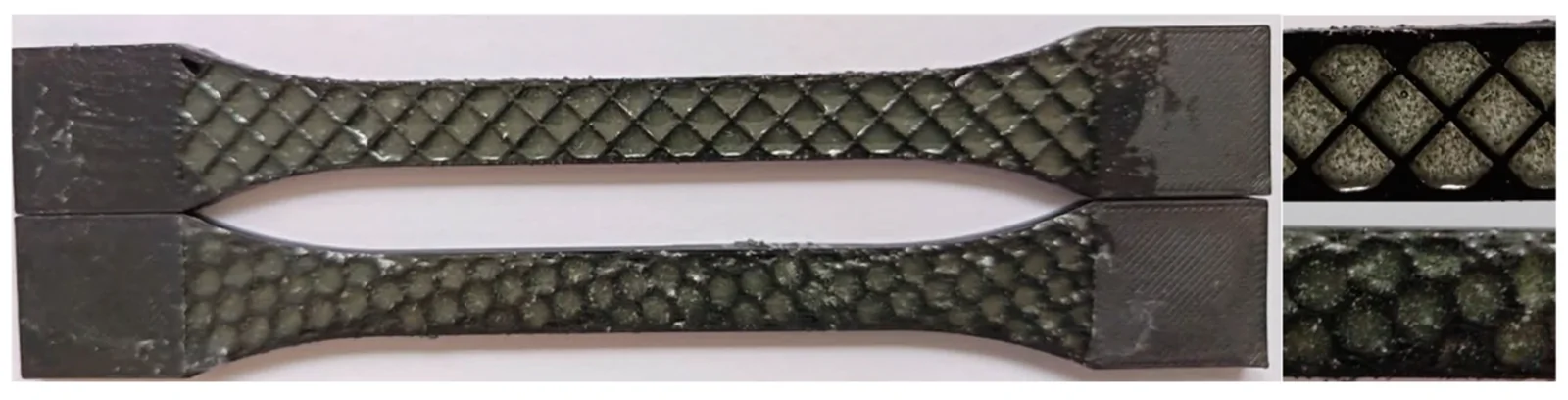
Universal tensile specimen: (**left**)—overall view; (**right**)—detail of the rectangular (grid) core (**top**) and honeycomb core (**bottom**).

The prepared composite mixture was manually applied to universal test specimens with a 3D-printed honeycomb core in hexagonal and rectangular shapes. Before filling, one side of the honeycomb core was sealed with adhesive tape to prevent the mixture from leaking during application. After filling, the test specimens were left to cure at room temperature for 72 h. After curing (Figure 4), the test specimens were prepared for mechanical testing. The specimens were tested in the as-printed condition without any additional surface preparation, such as sanding or polishing. The external surfaces retained the characteristic surface texture resulting from the FDM process, with visible deposited filament paths. No additional modification was applied to either the bottom or outer surfaces of the specimens.

**Figure 5:**
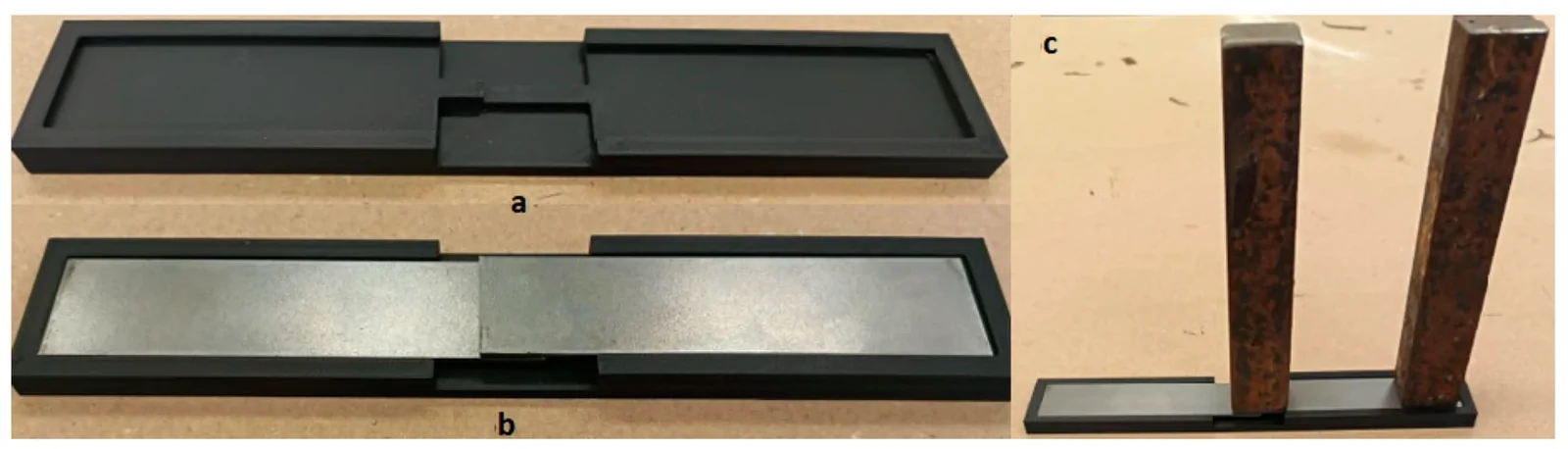
Fixture used for manufacturing single-lap adhesive joints: (**a**) fixture prior to adherend placement, (**b**) single-lap adhesive joints positioned in the fixture, (**c**) weighted joints during adhesive curing.

The same composite mixture was also used as an adhesive to produce lap joints. The mixture was applied between pre-prepared steel substrates, the preparation of which is described in Section 2.4. A constant thickness of the bonded layer was ensured using a 3D-printed bonding jig (Figure 5). In contrast, a constant adhesive layer thickness was ensured by loading the bonded joint with a weight of 716.4 g and a contact area of 25 × 25 mm, which exerted a constant load of 7.03 N during the curing of the adhesive.

**Figure 6:**
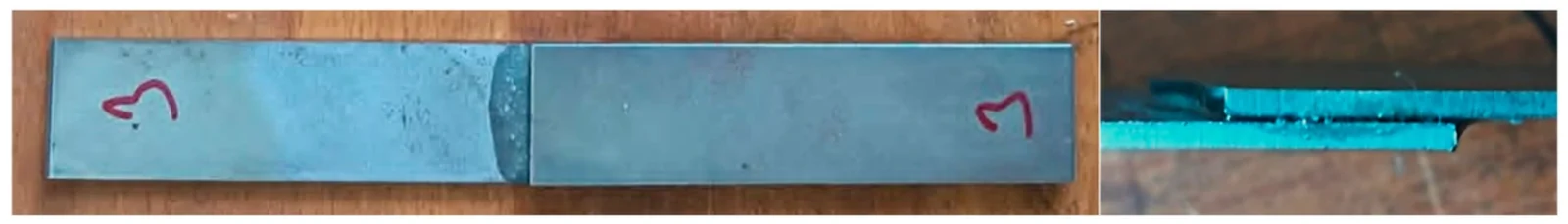
Single-lap joint specimen: (**left**)—overall view; (**right**)—detail of the bonded overlap.

The prepared lap joints (Figure 6) were cured at room temperature for 72 h and then prepared for mechanical testing.

### 2.6. Testing of Mechanical Properties

After curing, the universal test specimens with hexagonal and rectangular cores were clamped into the jaws of the LabTest 5.50 ST universal testing machine (LABORTECH s.r.o., Opava, Czech Republic) and subjected to a static tensile test at a crosshead speed of 10 mm·min^−1^. The measured data were recorded and subsequently evaluated using Test&Motion software (version 4.5.0.15, LABORTECH s.r.o., Opava, Czech Republic). The maximum tensile force, tensile strength, modulus of elasticity, and elongation at break were determined from the obtained data.

After curing, the laminated joints were subjected to a static tensile test on a LabTest 5.50 ST universal testing machine at a crosshead travel speed of 10 mm·min^−1^. The measured data were recorded and subsequently evaluated using Test&Motion software (Version 2026, Labortech s.r.o., Brno, Czech Republic). Based on the obtained data, the maximum force, shear strength of the bonded joint, and deformation characteristics of the individual modified adhesive systems were determined.

The measured data were then exported to Microsoft Excel (Microsoft 365; Microsoft Corporation, Redmond, WA, USA) and statistically analysed using Statistica software, version 14.0.0.15 (TIBCO Data Science Workbench; TIBCO Software Inc., Palo Alto, CA, USA). Linear regression analysis was performed to evaluate the relationship between PUF filler content and the investigated mechanical properties. The Pearson correlation coefficient (r), coefficient of determination (R^2^), and *p*-value were determined, with statistical significance assessed at *p* < 0.05.

### 2.7. SEM Analysis

The morphology of the samples was evaluated using a TESCAN VEGA 3 XMU scanning electron microscope (SEM) (TESCAN ORSAY HOLDING a.s., Brno, Czech Republic) at Jan Evangelista Purkyně University in Ústí nad Labem. Before SEM analysis, the samples were coated with a thin layer of gold using a Quorum Q150R ES Plus (Quorum Technologies, Laughton, United Kingdom).

SEM was used at several magnification levels (MAG) to characterise the microstructure of the recycled polyurethane filler and the fracture surfaces of the composite specimens. The individual SEM micrographs are arranged to enable a step-by-step evaluation of morphology, from an overall view to the detailed microstructure of the analysed areas.

The fracture surfaces analyzed by SEM were obtained directly from specimens fractured during the tensile tests and bonded-joint tests described above.

## 3. Results

### 3.1. Morphology of Recycled Polyurethane Filler

**Figure 7:**
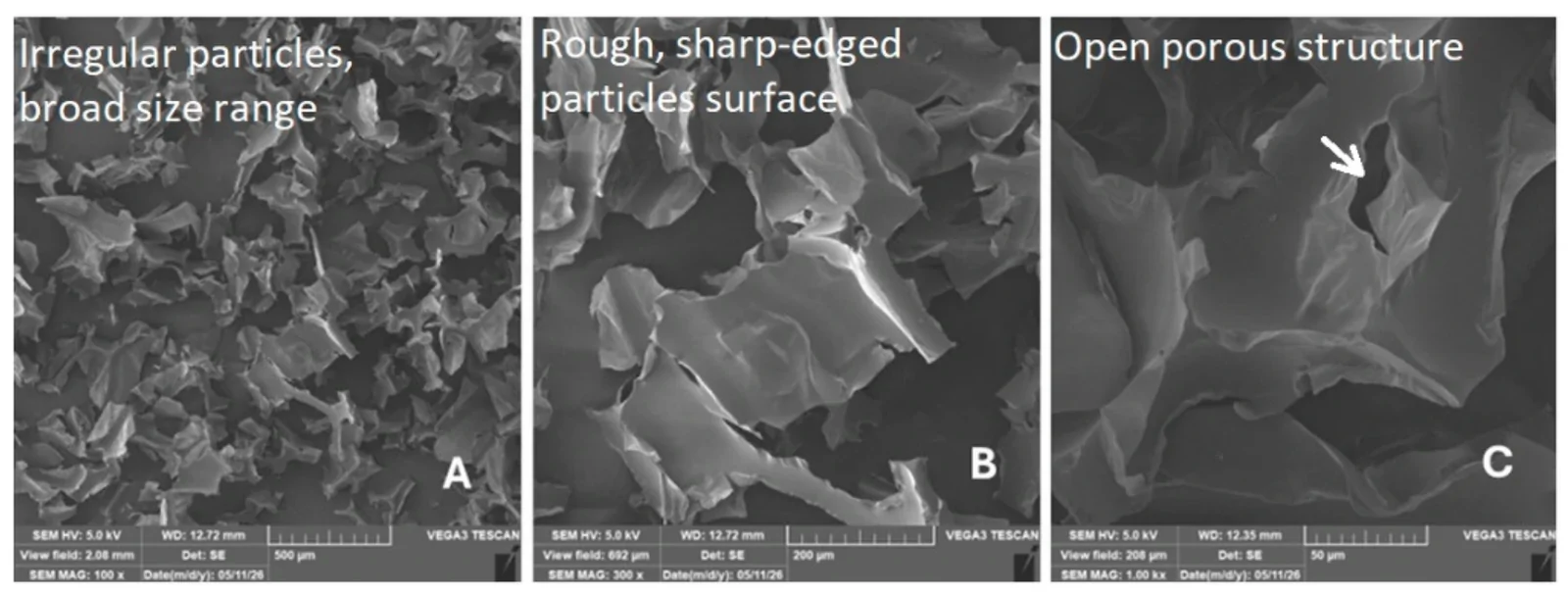
SEM micrographs of recycled crushed polyurethane foam (PUF35) obtained from sandwich panels with a density of 35 kg·m^−3^: (**A**) MAG 100×, (**B**) MAG 300, (**C**) MAG 1.00 k×.

**Figure 8:**
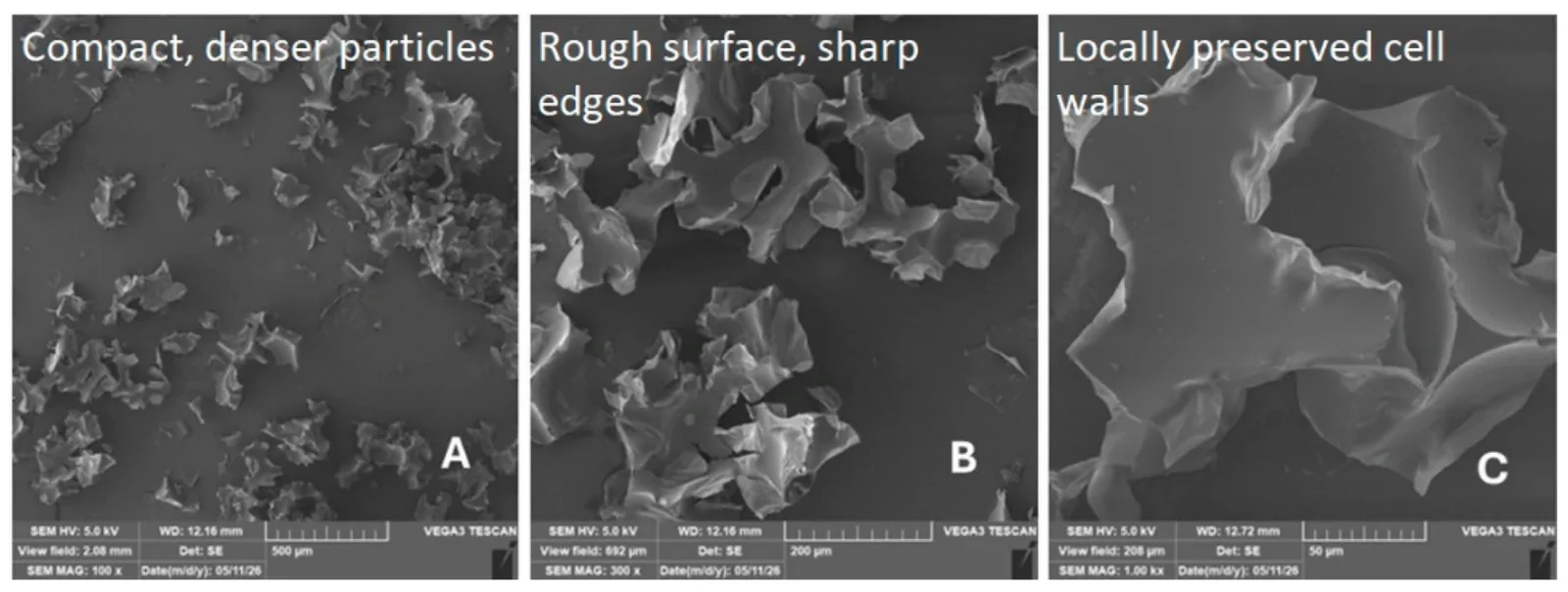
SEM micrographs of recycled crushed polyurethane foam (PUF60) obtained from sandwich panels with a bulk density of 60 kg·m^−3^: (**A**) MAG 100×, (**B**) MAG 300, (**C**) MAG 1.00 k×.

**Figure 9:**
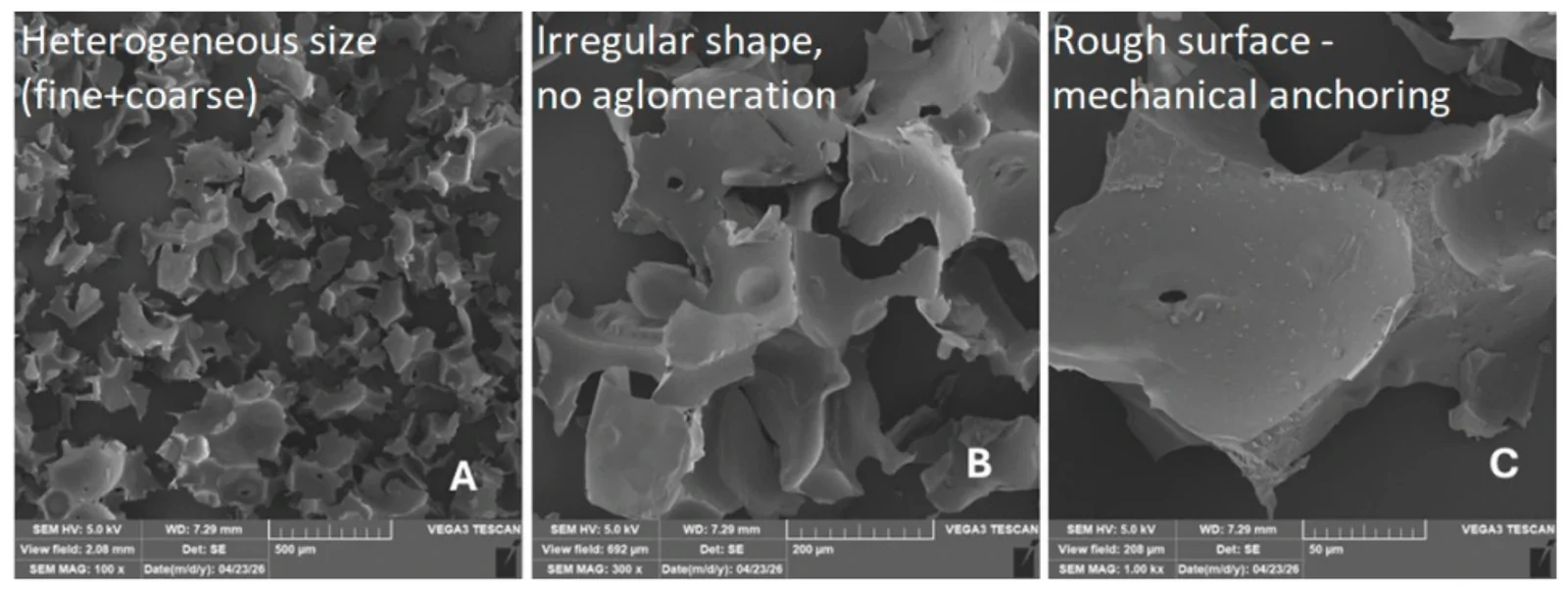
SEM micrographs of a mixture of recycled crushed polyurethane foam (PUF35/60) without sorting after the recycling process: (**A**) MAG 100×, (**B**) MAG 300, (**C**) MAG 1.00 k×.

Figure 7, Figure 8 and Figure 9 show SEM micrographs of recycled polyurethane fillers at progressively higher magnifications. Micrograph A provides an overview of particle morphology, size, and distribution, while micrographs B and C enable a more detailed evaluation of particle shape, surface characteristics, and local morphological features. In all analysed samples, mechanical crushing produced irregularly shaped particles with rough, rugged surfaces, sharp edges, and locally preserved fragments of the original cellular structure. SEM micrographs of PUF35 (Figure 7) show irregular particles with a relatively broad size distribution, including fragments with an open porous structure and pronounced surface roughness typical of mechanically crushed polymer foams. The PUF60 filler (Figure 8) exhibits similar morphology but appears more compact. The PUF35/60 filler (Figure 9) exhibits the greatest variability in particle size and shape. Despite the heterogeneous particle size, no significant agglomeration or excessively large particles were observed.

### 3.2. Rectangular Honeycomb Core (Grid)—Tensile Strength and Modulus of Elasticity

**Figure 10:**
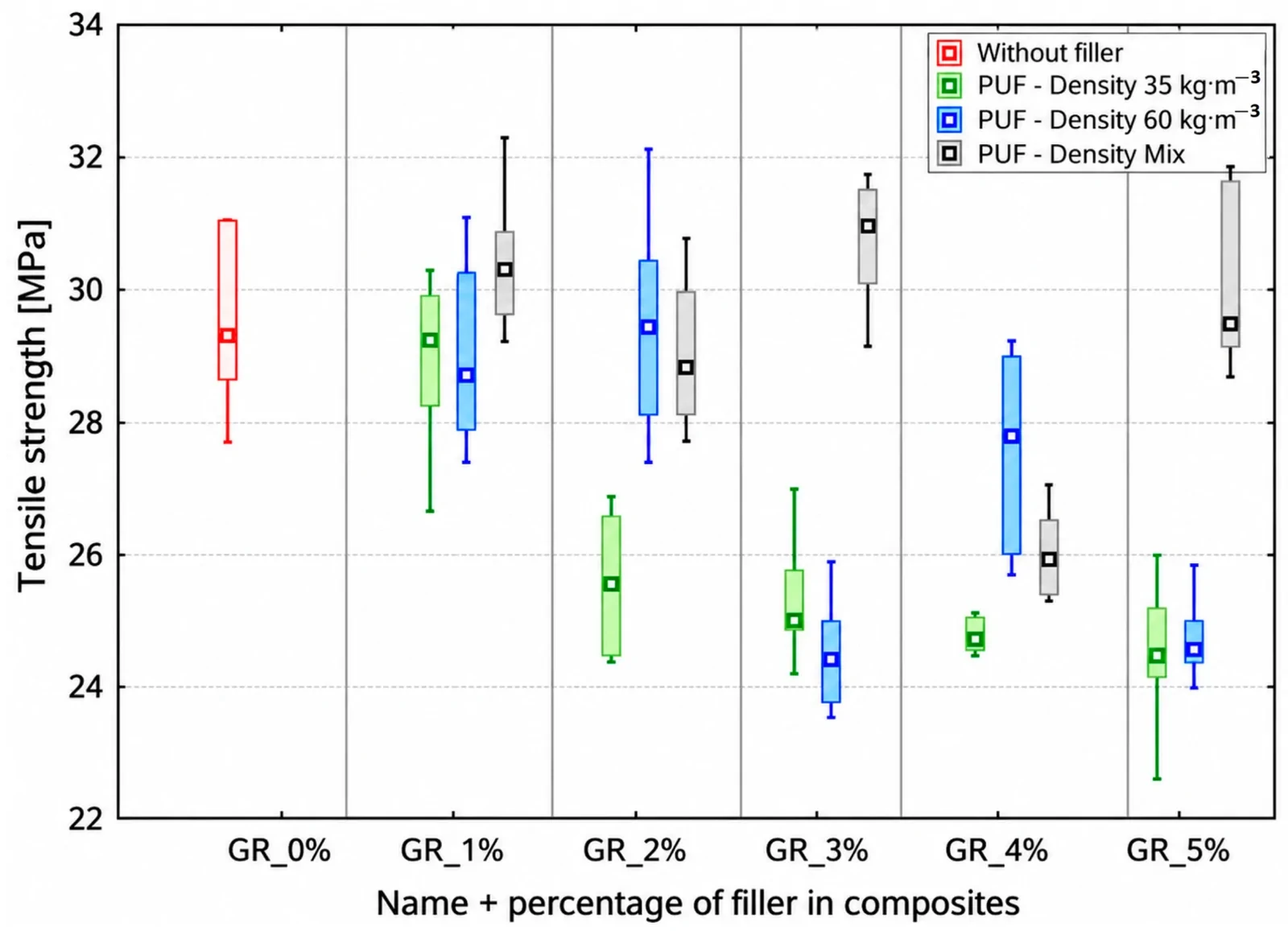
Tensile strength of the rectangular (grid) PLA core filled with epoxy resin containing different concentrations of recycled PUF.

The tensile strength results for composite materials with a rectangular honeycomb core modified with recycled PUF of various densities are shown in Figure 10. The reference composite without filler achieved an average tensile strength of 29.5 ± 1.4 MPa. The addition of 1 wt% recycled PUF maintained, or in some cases slightly increased, tensile strength across all tested variants. As the filler content increased, distinct differences among the individual types of recycled PUF became apparent. While composites containing PUF35 and PUF60 exhibited a gradual decrease in tensile strength at higher filler contents, the tensile strength of composites containing PUF35/60 remained at a similar level across the investigated concentration range. The results show that tensile strength was influenced by both filler content and recycled PUF type. The highest tensile strength was achieved with 3 wt% PUF35/60 (30.8 ± 1.0 MPa), representing an approximately 4.4% increase compared with the unfilled reference composite (29.5 ± 1.4 MPa), while the lowest values were recorded for 5 wt% PUF35 and PUF60.

Statistical analysis showed a strong and significant negative correlation between PUF content and tensile strength for PUF35 (r = −0.821, R^2^ = 0.674, *p* < 0.001) and a moderately strong and significant negative correlation for PUF60 (r = −0.663, R^2^ = 0.439, *p* < 0.001), indicating a significant decrease in tensile strength with increasing filler content. In contrast, PUF35/60 showed only a weak negative correlation (r = −0.209, R^2^ = 0.044, *p* = 0.222), which was not statistically significant, indicating a more stable tensile response across the investigated filler concentrations.

**Figure 11:**
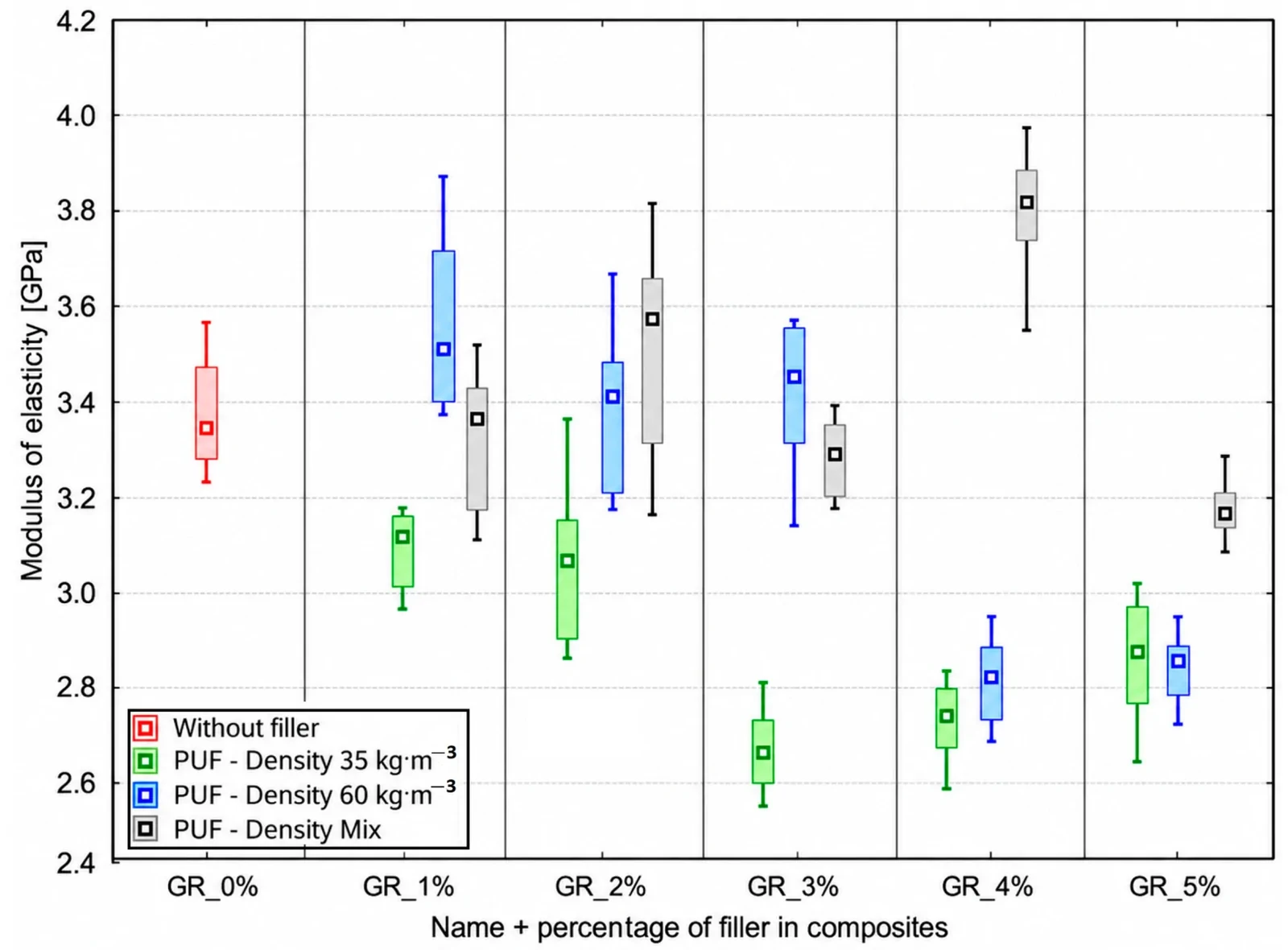
Modulus of elasticity of the rectangular (grid) PLA core filled with epoxy resin containing different concentrations of recycled PUF.

The modulus of elasticity results for composite materials with a rectangular honeycomb core modified with recycled PUF of various densities are shown in Figure 11. The reference sample without filler achieved an average modulus of elasticity of 3.37 ± 0.13 GPa. The addition of recycled PUF affected the modulus of elasticity depending on both filler density and content. Composite samples containing PUF35 exhibited a gradual decrease in the modulus of elasticity as the filler content increased, while a different trend was observed for composites containing PUF60 and PUF35/60. The highest modulus of elasticity was achieved for the composite containing 4 wt% PUF35/60 (3.80 ± 0.15 GPa), representing an approximately 12.8% increase compared with the unfilled reference sample (3.37 ± 0.13 GPa), while the lowest values were recorded for composites containing PUF35 at higher filler contents.

Statistical analysis of the modulus of elasticity showed a strong and statistically significant negative correlation with PUF content for both PUF35 (r = −0.731, R^2^ = 0.534, *p* < 0.001) and PUF60 (r = −0.727, R^2^ = 0.529, *p* < 0.001), confirming a decreasing linear trend with increasing filler content. In contrast, no significant linear relationship was observed for PUF35/60 (r = 0.041, R^2^ = 0.002, *p* = 0.811), indicating that the modulus of elasticity did not exhibit a systematic linear change with increasing filler content.

### 3.3. Hexagonal Honeycomb Core (Honeycomb)—Tensile Strength and Modulus of Elasticity

**Figure 12:**
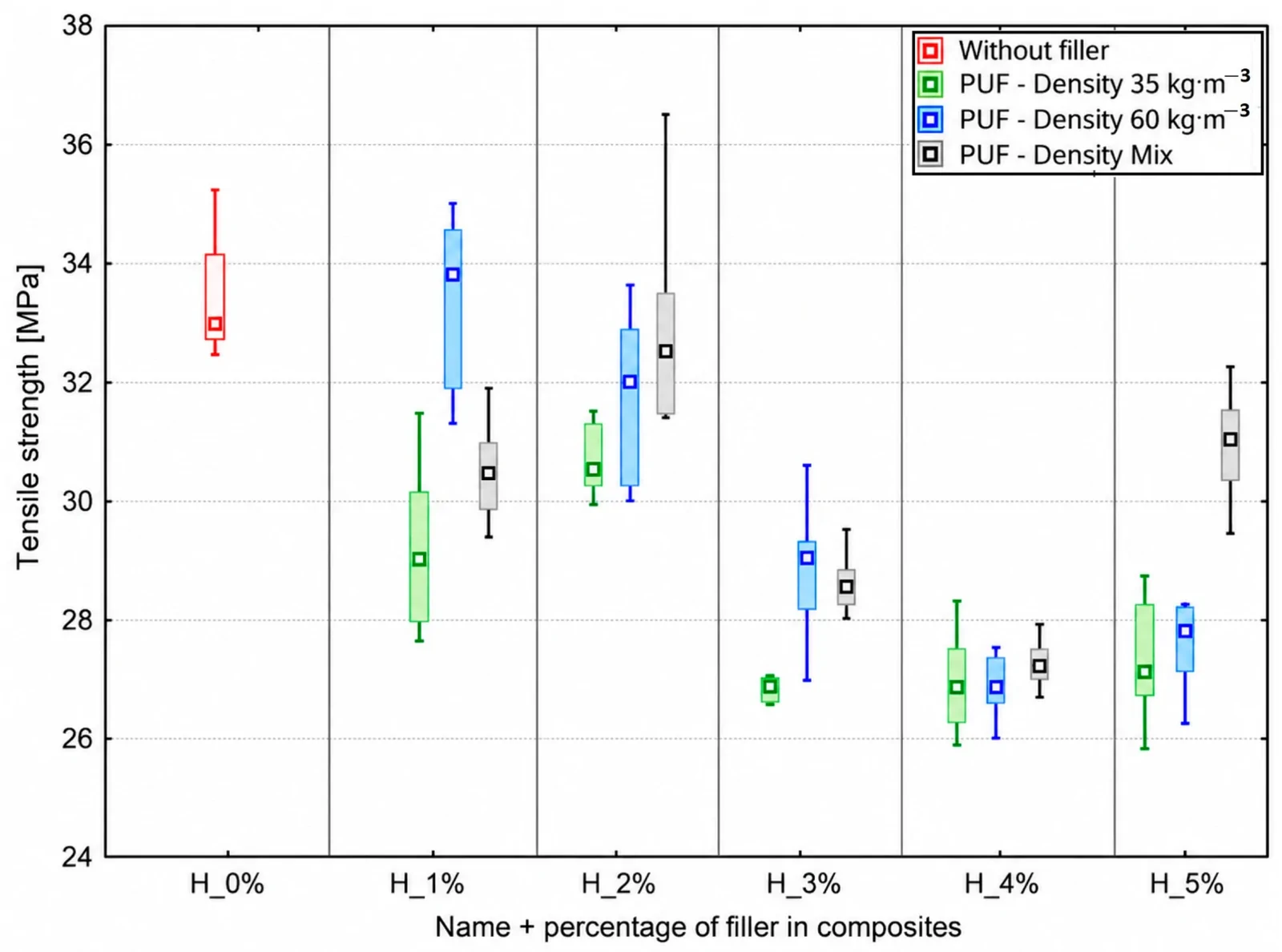
Tensile strength of the hexagonal honeycomb PLA core filled with epoxy resin containing different concentrations of recycled PUF.

The tensile strength results for composite materials with a hexagonal honeycomb core modified with recycled PUF of various densities are shown in Figure 12. The reference composite without filler achieved an average tensile strength of 32.9 ± 1.9 MPa. The addition of recycled PUF affected tensile strength depending on both filler density and content. The highest tensile strength was achieved in composites containing 1 wt% PUF60 (33.4 ± 1.1 MPa), corresponding to an approximately 1.5% increase compared with the unfilled reference composite (32.9 ± 1.9 MPa). As the filler content increased beyond 2 wt%, a gradual decrease in tensile strength was observed for all tested variants, with the lowest values recorded for composites containing 4 wt% PUF35 and PUF60.

Statistical analysis showed a strong and statistically significant negative correlation between PUF content and tensile strength for PUF35 (r = −0.790, R^2^ = 0.623, *p* < 0.001) and a very strong and statistically significant negative correlation for PUF60 (r = −0.875, R^2^ = 0.765, *p* < 0.001). PUF35/60 also exhibited a moderate and statistically significant negative correlation (r = −0.533, R^2^ = 0.284, and *p* < 0.001).

**Figure 13:**
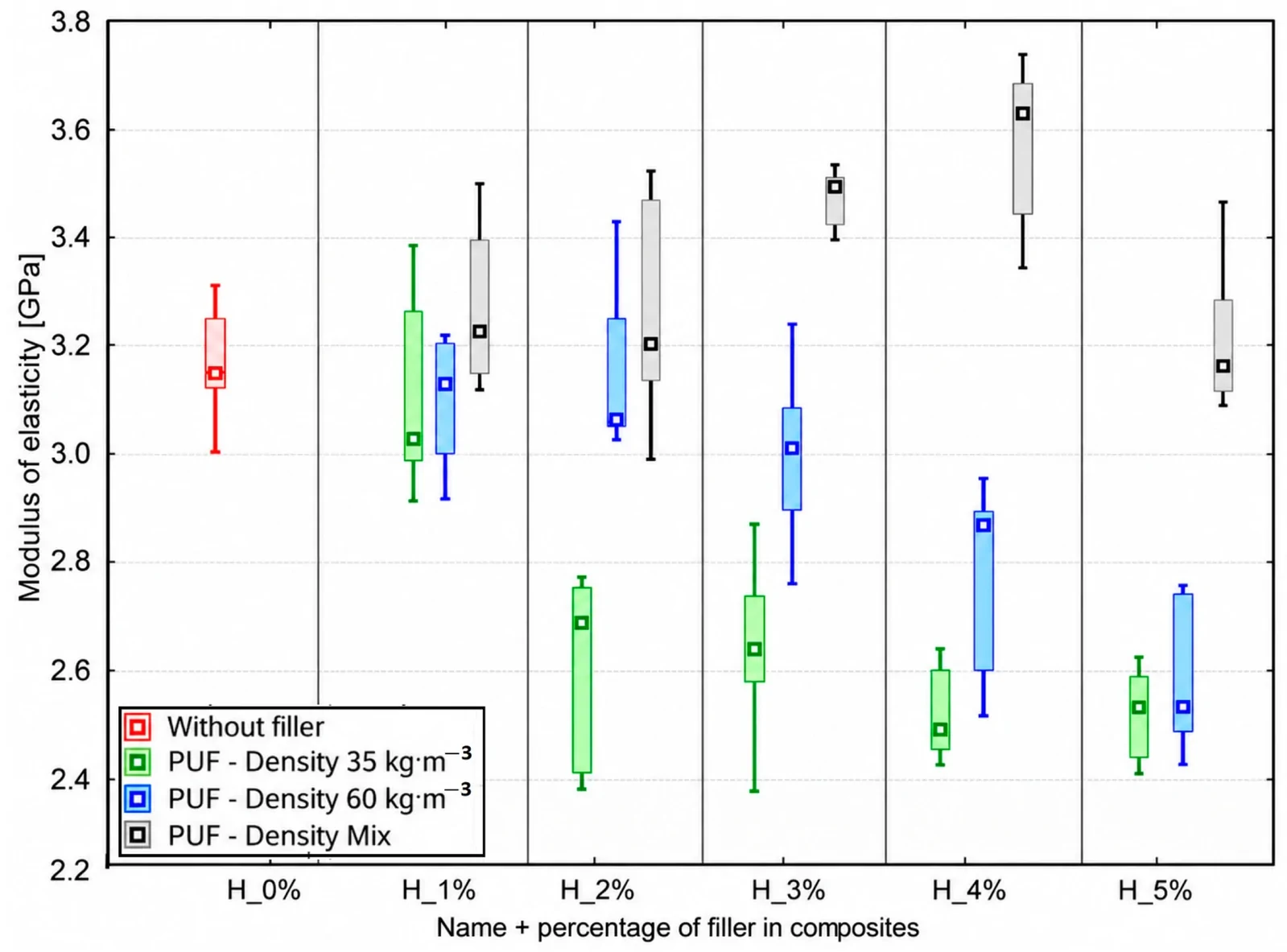
Modulus of elasticity of the hexagonal honeycomb PLA core filled with epoxy resin containing different concentrations of recycled PUF.

The results for the modulus of elasticity of composite materials with a hexagonal honeycomb core modified with recycled PUF of various densities are shown in Figure 13. The reference composite without filler achieved an average modulus of elasticity of 3.16 ± 0.13 GPa. The addition of recycled PUF affected the modulus of elasticity depending on both filler density and content. The highest modulus of elasticity was achieved for PUF35/60, with the maximum value recorded at 4 wt% filler (3.60 ± 0.19 GPa), representing an approximately 13.9% increase compared with the unfilled reference composite (3.16 ± 0.13 GPa). In contrast, composites modified with PUF35 exhibited predominantly lower modulus values than the reference composite as the filler content increased, while composites containing PUF60 showed a different trend depending on filler content.

Statistical analysis showed a strong and statistically significant negative correlation between PUF content and the modulus of elasticity for PUF35 (r = −0.812, R^2^ = 0.659, *p* < 0.001) and PUF60 (r = −0.774, R^2^ = 0.599, and *p* < 0.001). In contrast, PUF35/60 exhibited a weak positive but statistically significant correlation (r = 0.342, R^2^ = 0.117, and *p* = 0.041).

### 3.4. Microstructural Analysis of Fracture Surfaces (SEM)

**Figure 14:**
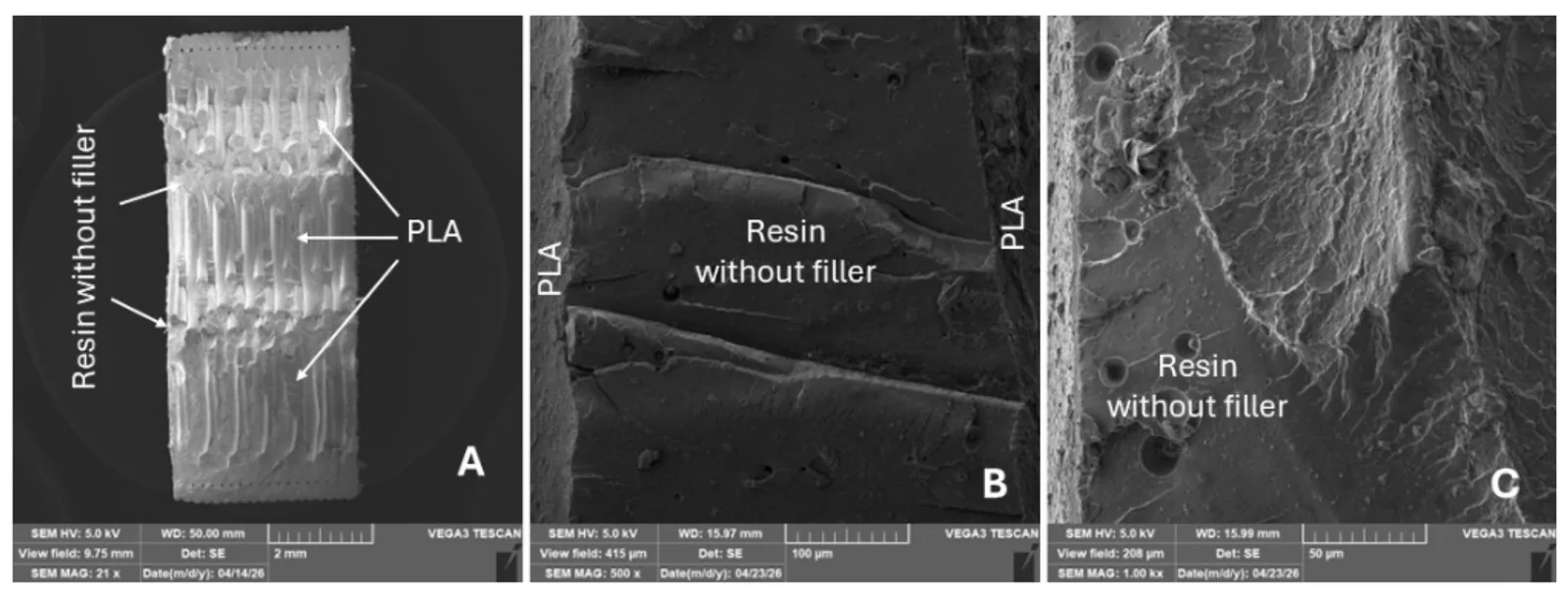
SEM micrographs of the fracture surface of the reference sample (PLA honeycomb core filled with a pure epoxy matrix): (**A**) overview of the fracture surface (MAG 21×); (**B**) detail of the phase interface between the 3D-printed PLA honeycomb and the epoxy matrix (MAG 500×); (**C**) close-up of the pure epoxy matrix at higher magnification (MAG 1.00 k×).

**Figure 15:**
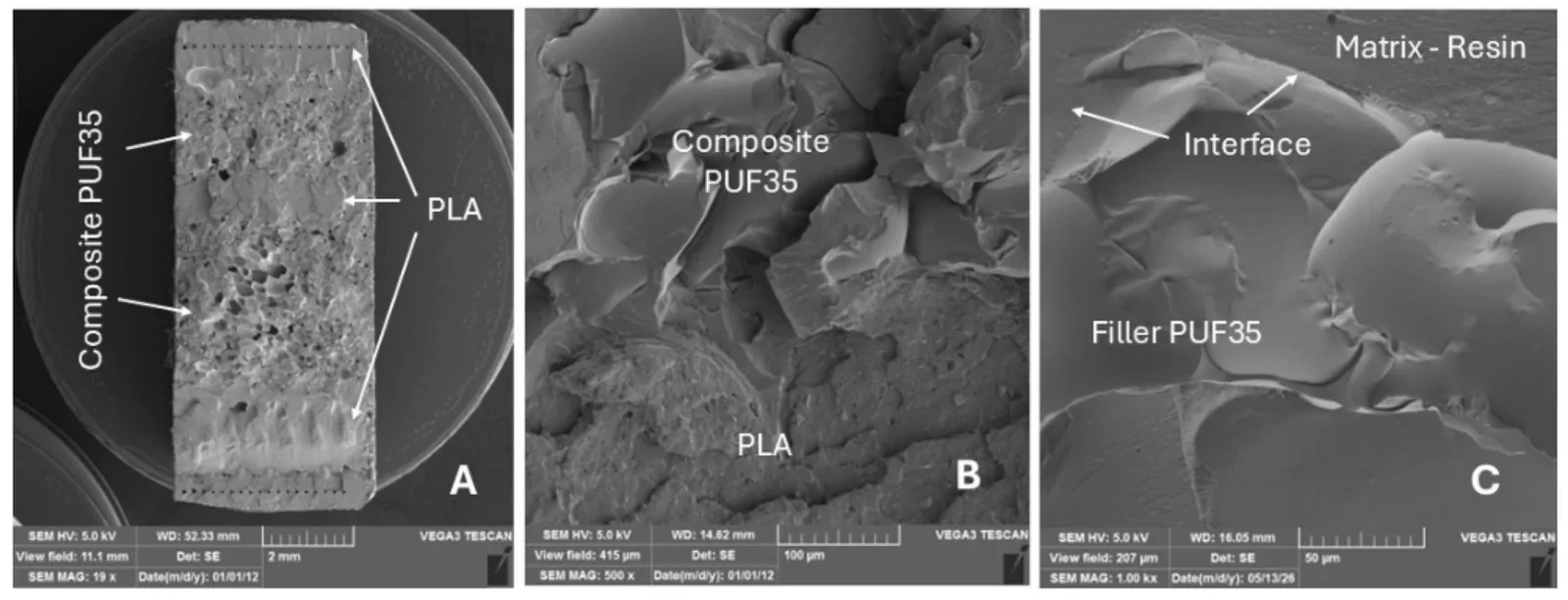
SEM micrographs of the fracture surface of a composite sample with PUF35 filler: (**A**) overview of the fracture surface (MAG 19×); (**B**) detail of the phase interface between the PLA honeycomb and the composite filler (MAG 500×); (**C**) microstructure of the composite layer showing the dispersion of PUF35 particles in the epoxy matrix (MAG 1.00 k×).

**Figure 16:**
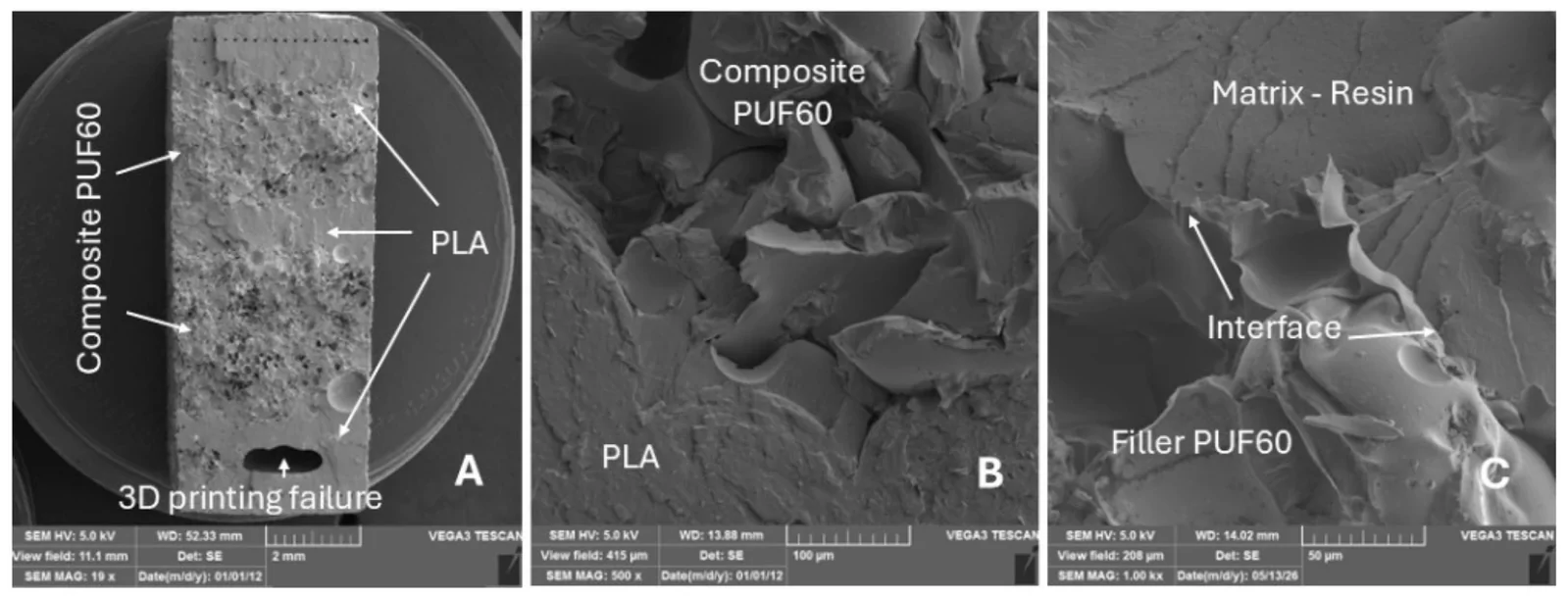
SEM micrographs of the fracture surface of a composite sample with PUF60 filler: (**A**) overview of the structure (MAG 19×); (**B**) detail of the phase interface between the PLA honeycomb and the composite filler (MAG 500×); (**C**) detail of the interaction between PUF60 particles and the epoxy matrix and the cohesive nature of the fracture (MAG 1.00 k×).

**Figure 17:**
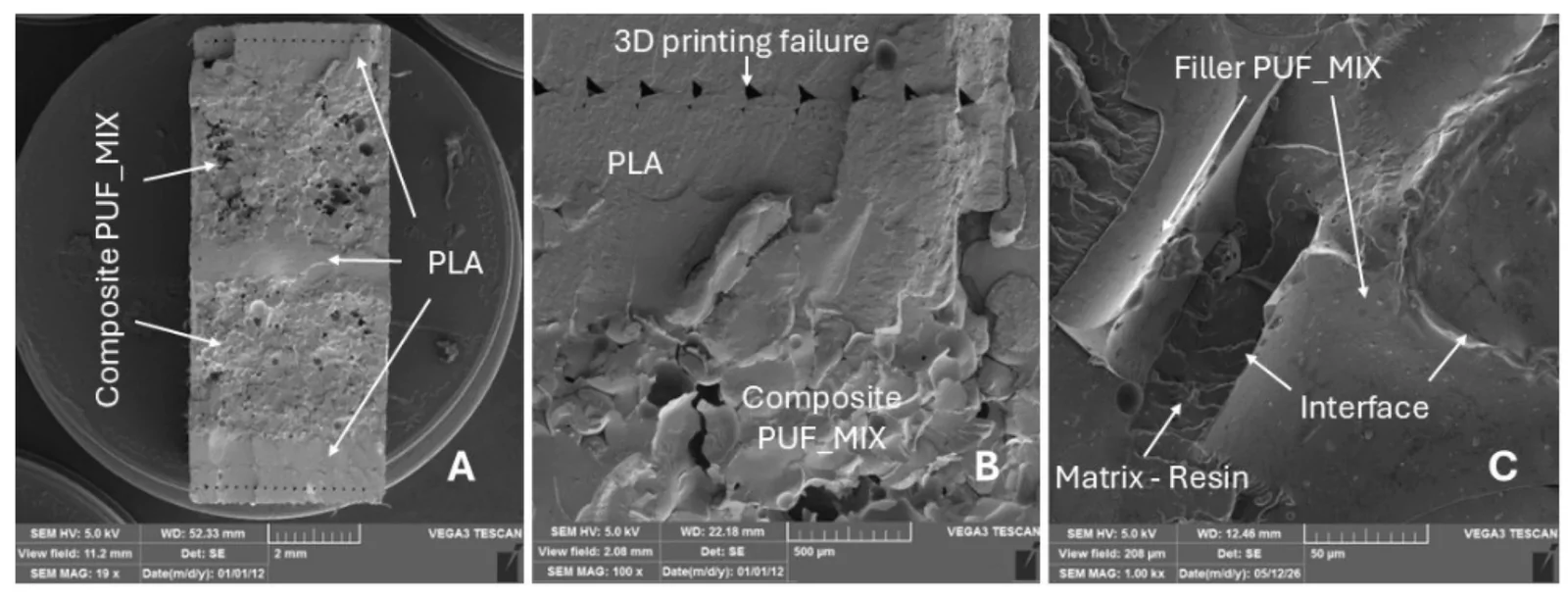
SEM micrographs of the fracture surface of a composite sample with PUF35/60 hybrid filler: (**A**) overview of the fracture surface (MAG 19×); (**B**) detail of the phase interface between the PLA honeycomb and the composite filler showing a printing defect (3D printing failure) (MAG 100×); (**C**) detail of the uniform distribution of the PUF35/60 particle/filler mixture in the epoxy matrix (MAG 1.00 k×).

Figure 14, Figure 15, Figure 16 and Figure 17 show representative SEM micrographs of fracture surfaces after mechanical loading using the rectangular honeycomb core. In each figure, micrographs A, B, and C show the overall fracture surface, PLA–epoxy interface, and higher-magnification details of filler dispersion and failure mechanisms, respectively.

The reference sample (Figure 14) shows a compact epoxy matrix with good adhesion to the PLA core and predominantly cohesive failure. Similarly, PUF35 and PUF60 (Figure 15 and Figure 16) show particles firmly embedded in the matrix without significant pull-out or interfacial separation. PUF35/60 (Figure 17) also exhibits uniform particle dispersion and a continuous PLA–composite interface without significant defects.

Compared with the reference sample, PUF-containing composites (Figure 15, Figure 16 and Figure 17) exhibit rougher fracture surfaces and more complex crack paths. Local voids and irregularities were observed mainly in the vicinity of the porous PUF particles; however, no significant porosity disrupting the continuity of the composite layer was observed.

SEM analysis also revealed the characteristic layered FDM structure of the PLA core without delamination between the deposition paths. Close contact between the epoxy and PLA surfaces and penetration of the epoxy into local FDM surface irregularities were observed. Although local printing defects were visible, particularly in Figure 16 and Figure 17, no associated crack initiation or interfacial separation was observed.

### 3.5. Overlapped Joints—Adhesive Bond Strength and Modulus of Elasticity

**Figure 18:**
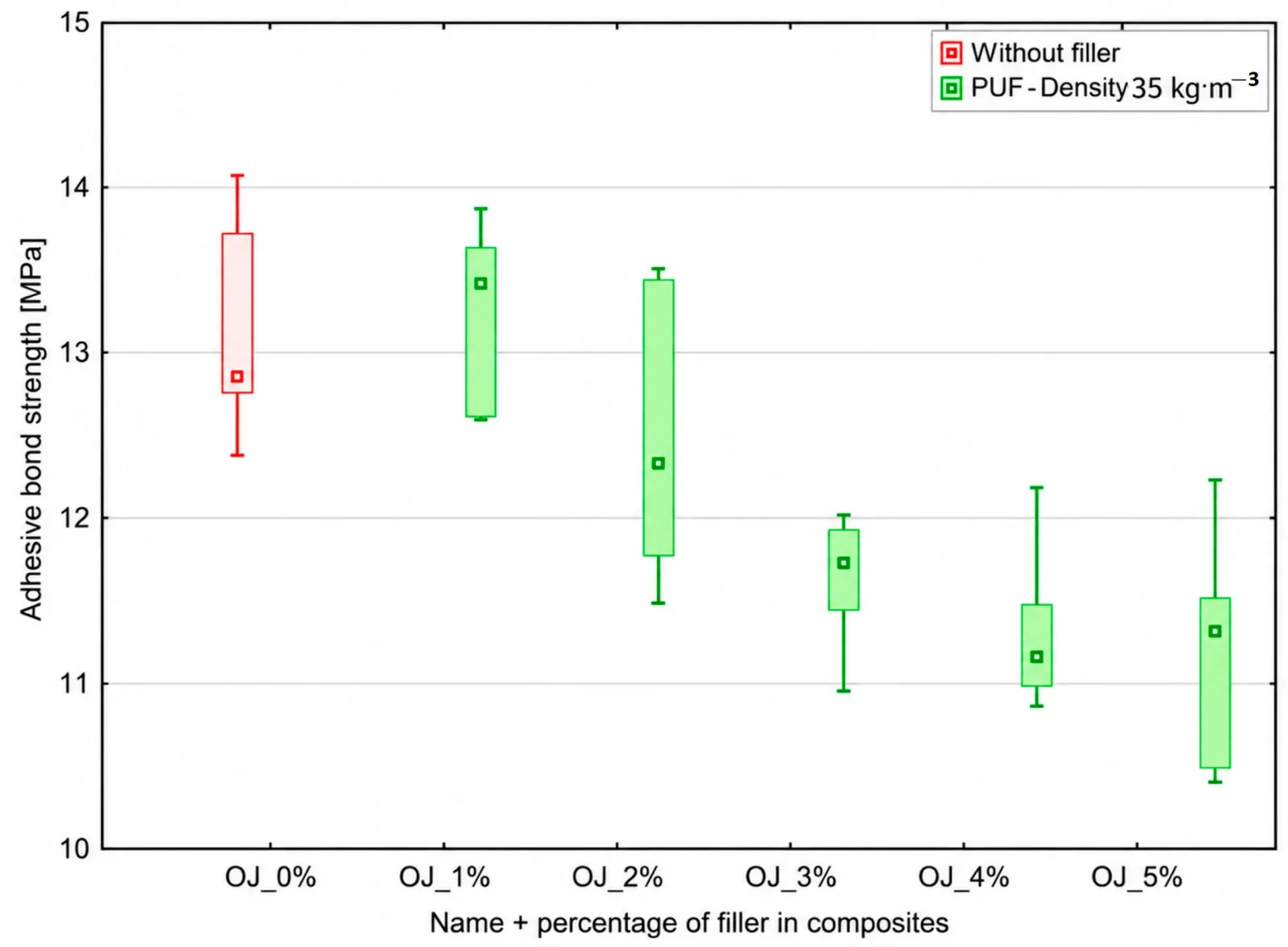
Adhesive bond strength of single-lap joints modified with 1–5 wt% recycled PUF35 in the epoxy adhesive.

The results of the tensile adhesive bond strength of lap joints modified with recycled PUF35 are shown in Figure 18. The reference joint without filler achieved an average adhesive bond strength of 13.1 ± 0.7 MPa. The addition of recycled PUF affected the adhesive bond strength depending on the filler content. The highest adhesive bond strength of the modified joints was recorded at a recycled PUF content of 1 wt% (13.3 ± 0.5 MPa), corresponding to an approximately 1.5% increase compared with the reference joint without filler (13.1 ± 0.7 MPa), while the lowest value was achieved at 5 wt% (11.2 ± 0.7 MPa), representing an approximately 14.5% decrease compared with the reference joint.

As the filler content increased, the adhesive bond strength gradually decreased. Statistical analysis showed a strong and statistically significant negative correlation between PUF35 content and shear strength (r = −0.788, R^2^ = 0.621, *p* < 0.001).

**Figure 19:**
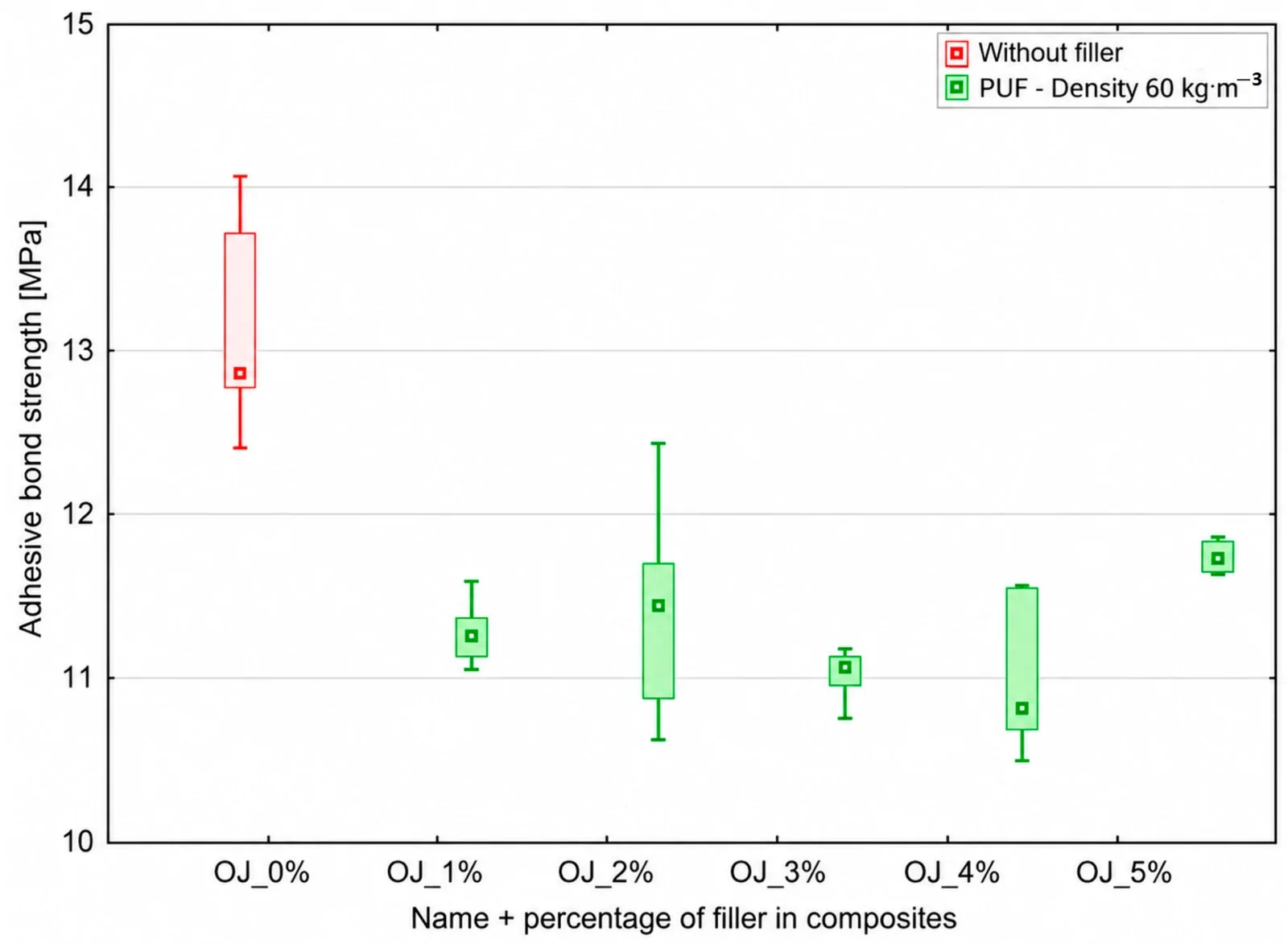
Adhesive bond strength of single-lap joints modified with 1–5 wt% recycled PUF60 in the epoxy adhesive.

The results of adhesive bond strength for lap joints modified with recycled PUF60 are shown in Figure 19. The reference joint without filler achieved an average adhesive bond strength of 13.1 ± 0.7 MPa. The addition of recycled PUF affected the adhesive bond strength depending on the filler content. The highest adhesive bond strength among the modified joints was recorded at a recycled PUF content of 5 wt% (11.7 ± 0.1 MPa), representing an approximately 10.7% decrease compared with the reference joint. The lowest value was achieved at a content of 4 wt% (11.0 ± 0.5 MPa), corresponding to an approximately 16.0% decrease compared with the reference joint. No clear trend in adhesive bond strength was observed within the concentration range studied.

Statistical analysis showed a moderate and statistically significant negative correlation between PUF60 content and shear strength (r = −0.473, R^2^ = 0.224, and *p* = 0.0036).

**Figure 20:**
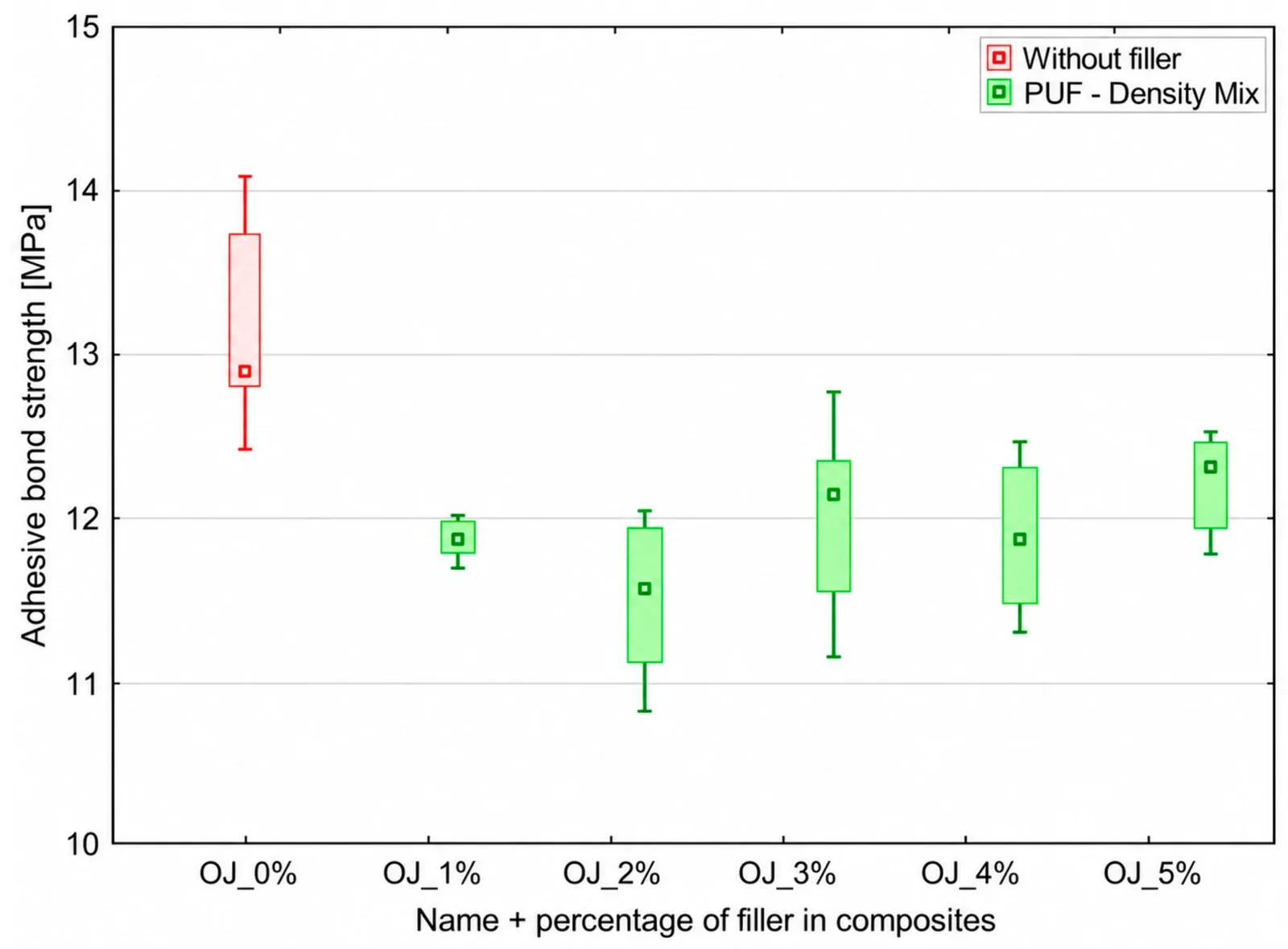
Adhesive bond strength of single-lap joints modified with 1–5 wt% recycled PUF35/60 in the epoxy adhesive.

The adhesive bond strength results for lap joints modified with recycled PUF35/60 are shown in Figure 20. The reference joint without filler achieved an average adhesive bond strength of 13.1 ± 0.7 MPa. The addition of recycled PUF35/60 affected the adhesive bond strength depending on the filler content. The highest adhesive bond strength among the modified joints was recorded at a PUF35/60 content of 5 wt% (12.2 ± 0.3 MPa), representing an approximately 6.9% decrease compared with the reference joint. The lowest value was achieved at a content of 2 wt% (11.5 ± 0.5 MPa), corresponding to an approximately 12.2% decrease compared with the reference joint. No clear trend in adhesive bond strength was observed within the concentration range studied.

Statistical analysis showed a weak negative correlation between PUF35/60 content and shear strength (r = −0.296, R^2^ = 0.088, and *p* = 0.0794); however, this relationship was not statistically significant.

**Figure 21:**
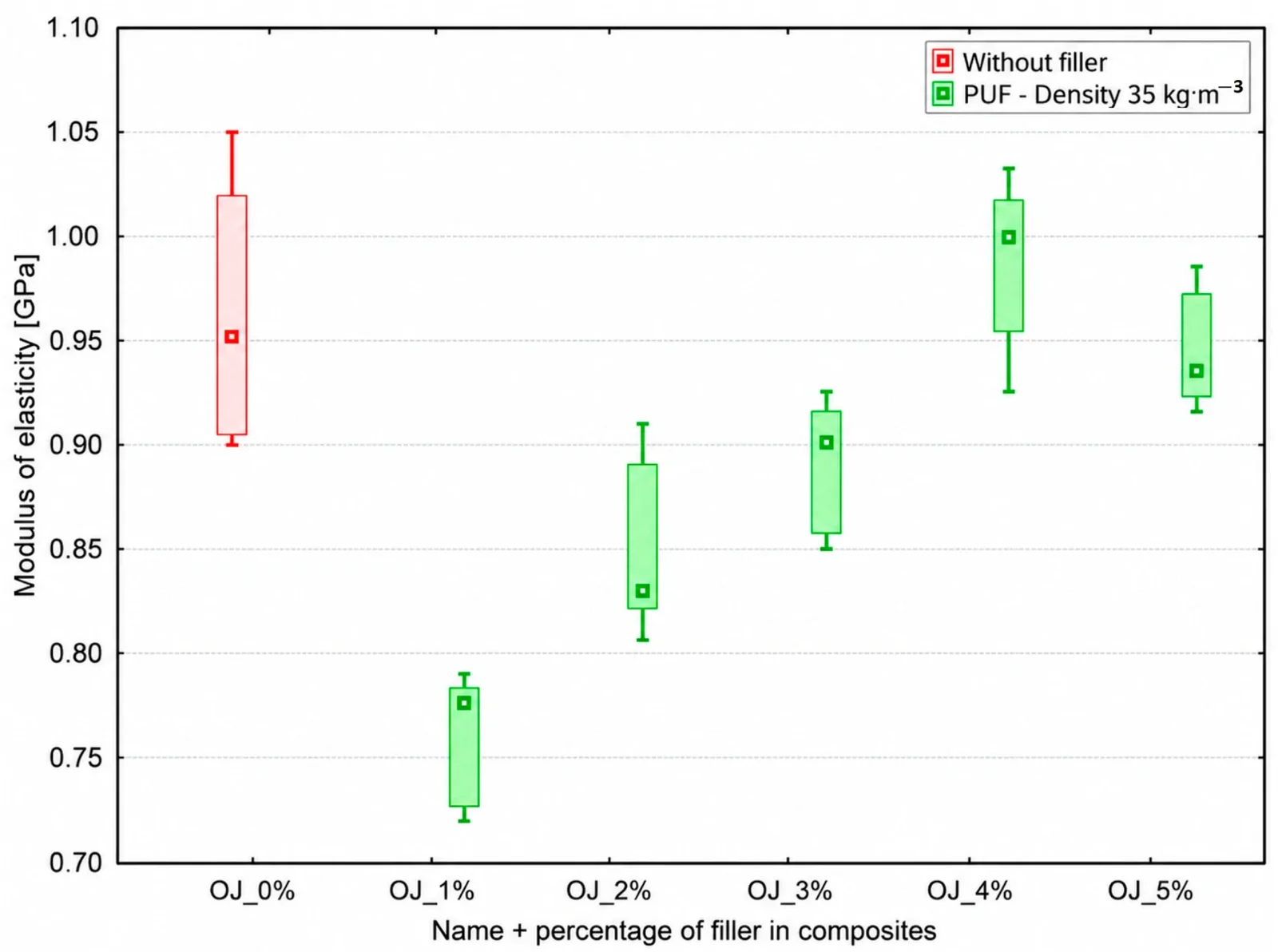
Modulus of elasticity of single-lap joints modified with 1–5 wt% recycled PUF35 in the epoxy adhesive.

The results for the modulus of elasticity of lap joints modified with recycled PUF35 are shown in Figure 21. The reference joint without filler achieved an average modulus of elasticity of 0.96 ± 0.06 GPa. The addition of recycled PUF35 affected the modulus of elasticity, with the effect depending on the filler content. The lowest modulus of elasticity was achieved in joints containing 1 wt% recycled PUF35 (0.76 ± 0.03 GPa), representing an approximately 20.8% decrease compared with the reference joint. In contrast, the highest modulus of elasticity among the modified joints was recorded at a content of 4 wt% (0.99 ± 0.04 GPa), representing an approximately 3.1% increase compared with the reference joint. Following a significant decrease in the modulus of elasticity at 1 wt% filler content, it gradually increased with increasing recycled PUF35 content, although a slight decrease was observed at 5 wt%.

Statistical analysis showed a weak positive correlation between PUF35 content and the modulus of elasticity (r = 0.309, R^2^ = 0.096, and *p* = 0.0665). However, this relationship was not statistically significant.

**Figure 22:**
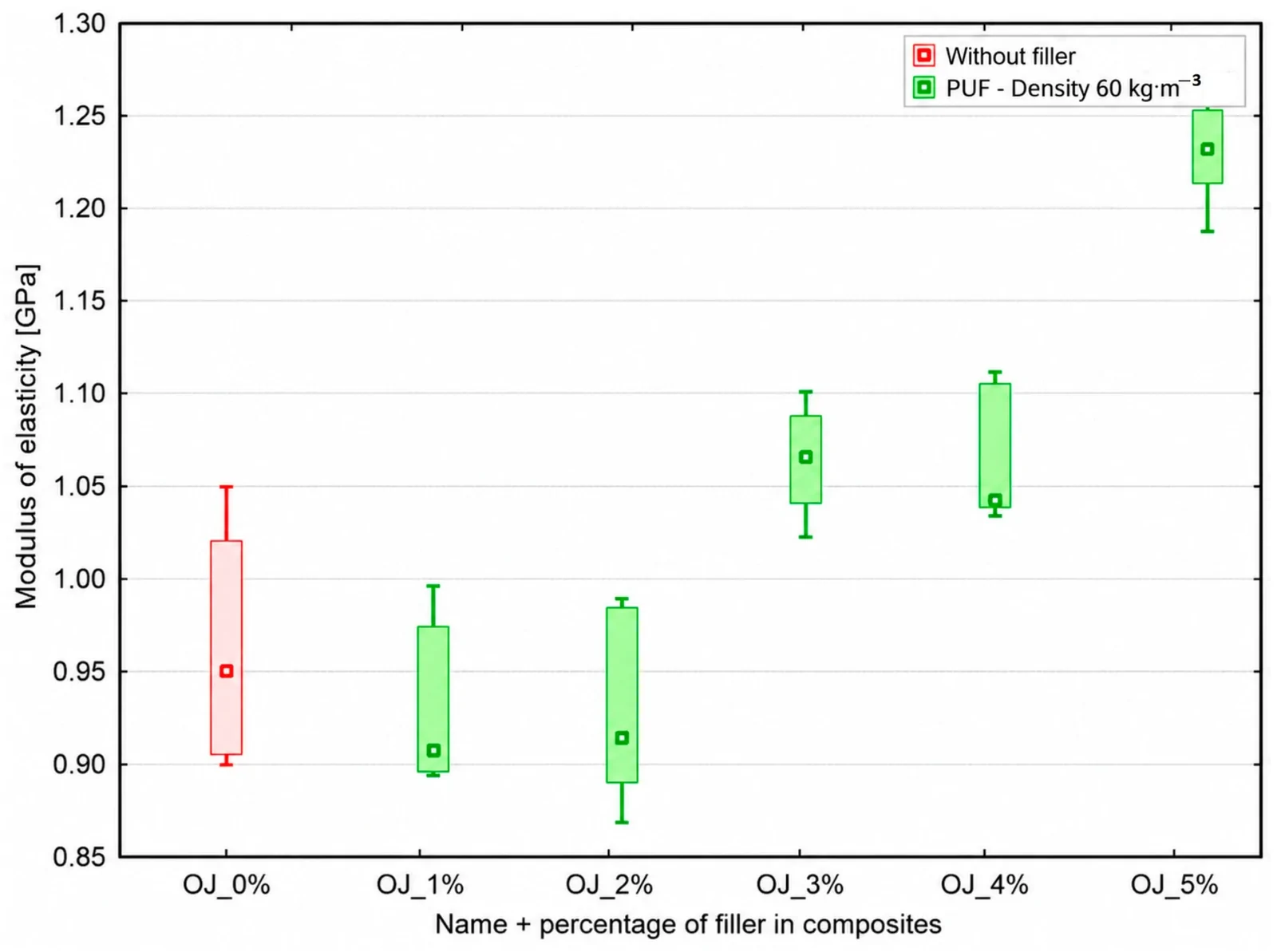
Modulus of elasticity of single-lap joints modified with 1–5 wt% recycled PUF60 in the epoxy adhesive.

The results for the modulus of elasticity of lap joints modified with recycled PUF60 are shown in Figure 22. The reference joint without filler achieved an average modulus of elasticity of 0.96 ± 0.06 GPa. The addition of recycled PUF60 affected the modulus of elasticity, with the effect depending on the filler content. The lowest modulus of elasticity was achieved for joints containing 2 wt% recycled PUF60 (0.93 ± 0.05 GPa), representing an approximately 3.1% decrease compared with the reference joint. In contrast, the highest modulus of elasticity among the modified joints was recorded at a content of 5 wt% (1.23 ± 0.34 GPa), representing an approximately 28.1% increase compared with the reference joint. Following a slight decrease in the modulus of elasticity at filler contents of 1 and 2 wt%, a significant increase was observed with increasing recycled PUF60 content.

Statistical analysis showed a strong and statistically significant positive correlation between PUF60 content and the modulus of elasticity (r = 0.685, R^2^ = 0.470, and *p* < 0.001).

**Figure 23:**
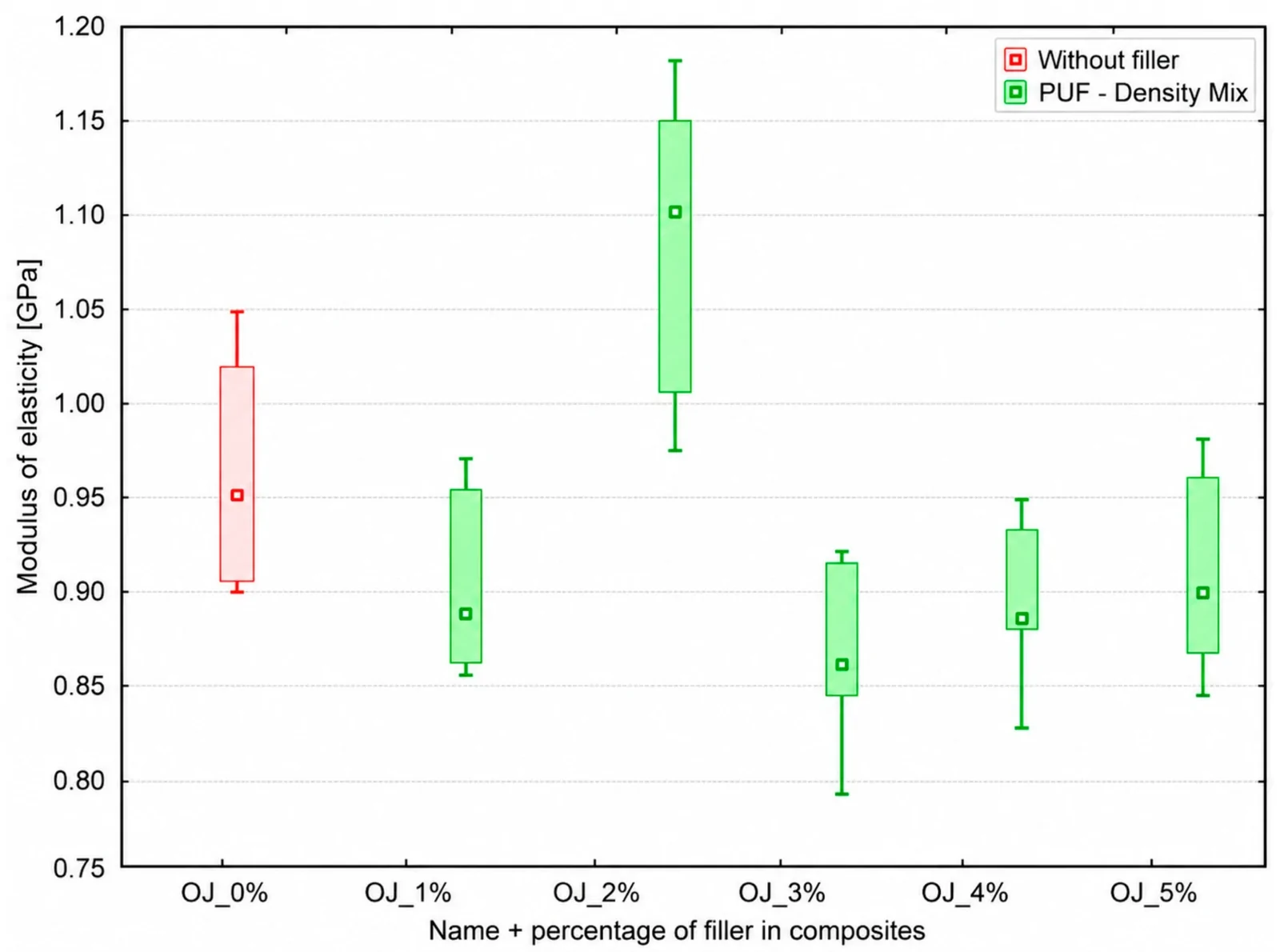
Modulus of elasticity of single-lap joints modified with 1–5 wt% recycled PUF35/60 in the epoxy adhesive.

The results for the modulus of elasticity of lap joints modified with recycled PUF35/60 are shown in Figure 23. The reference joint without filler achieved an average modulus of elasticity of 0.96 ± 0.06 GPa. The addition of PUF35/60 affected the modulus of elasticity, with the effect depending on the filler content. The highest modulus of elasticity was achieved in joints containing 2 wt% PUF35/60 (1.09 ± 0.08 GPa), representing an approximately 13.5% increase compared with the reference joint. In contrast, the lowest modulus of elasticity was recorded at a content of 3 wt% (0.86 ± 0.05 GPa), representing an approximately 10.4% decrease compared with the reference joint. An increase in the modulus of elasticity at a filler content of 2 wt% was followed by a decrease at 3 wt%, with a gradual increase again observed at higher PUF35/60 contents.

Statistical analysis showed a negligible negative correlation between PUF35/60 content and the modulus of elasticity (r = −0.046, R^2^ = 0.002, and *p* = 0.791), which was not statistically significant.

### 3.6. SEM Analysis of Overlapped Joints

**Figure 24:**
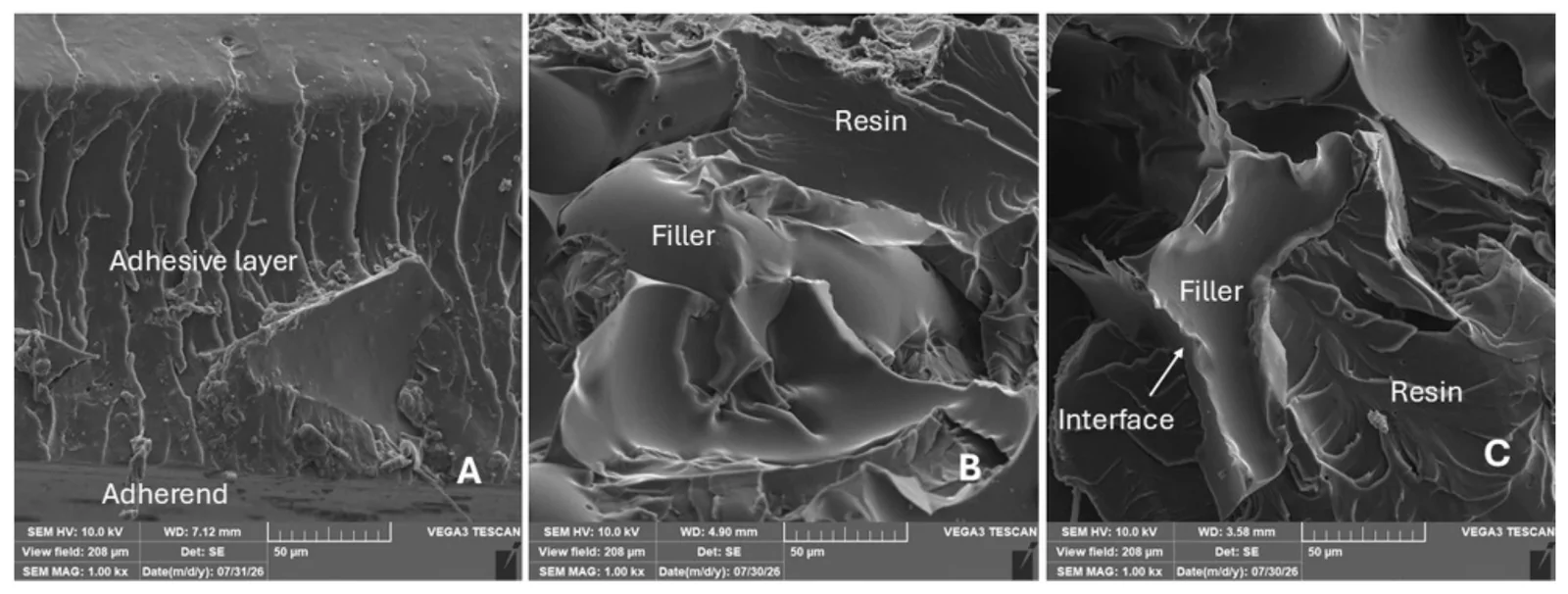
SEM micrographs of the fracture surface of a bonded steel joint: (**A**) an overall view of the adhesive–cohesive fracture (ACF) showing the interaction between the adhesive layer and the steel substrate (MAG 1.00 k×); (**B**) the interaction between the matrix and the filler (MAG 1.00 k×); (**C**) the interaction between the matrix and the filler (MAG 1.00 k×).

The fracture surfaces of the bonded joints exhibited a combined type of failure, specifically adhesive–cohesive fracture (ACF), as shown in Figure 24A. The filler exhibited close contact with both the polymer matrix (Figure 24B,C) and the bonded steel adherend (Figure 24A). The details in Figure 24B,C also show differences in the morphology of the filler particles. Furthermore, the nature of the fracture surfaces did not change significantly after the filler was incorporated into the matrix.

**Figure 25:**
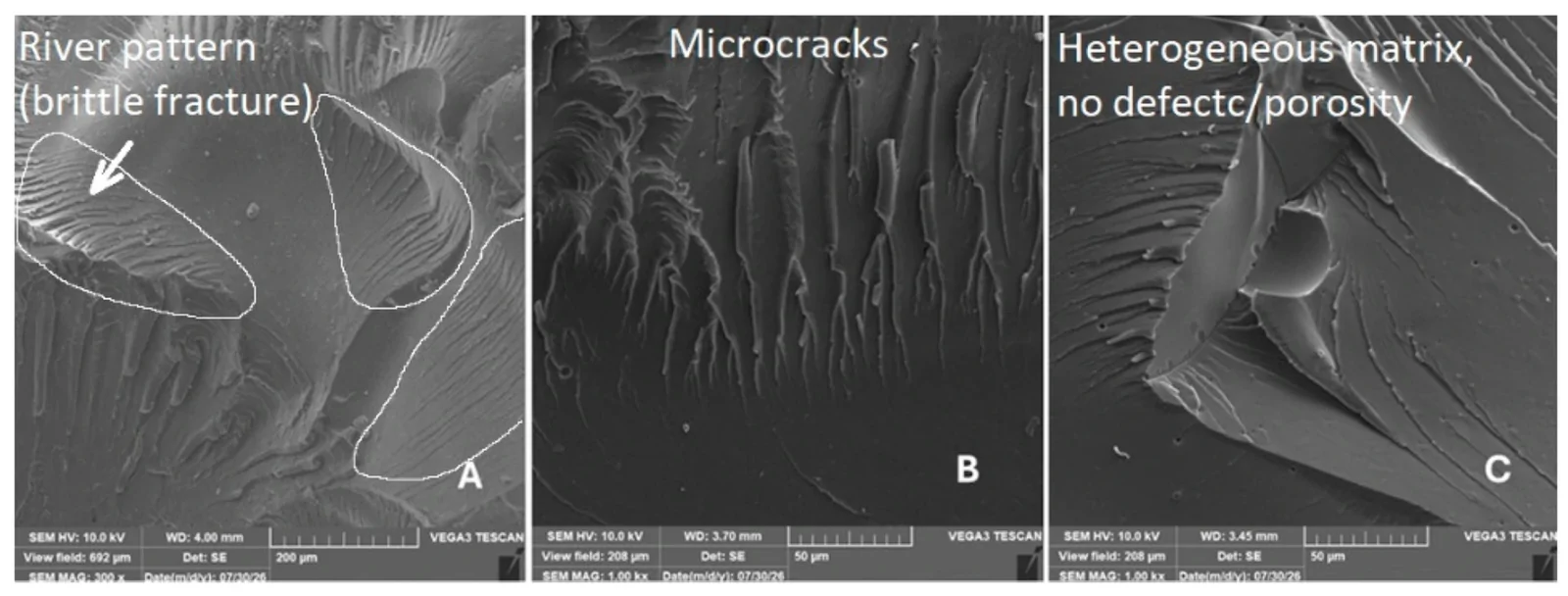
SEM micrographs of the fracture surface of a polymer matrix without filler: (**A**) a fracture surface parallel to the direction of loading, showing a smooth matrix texture with crack propagation lines (MAG 300×); (**B**) detail of the fracture surface perpendicular to the direction of loading, showing clear signs of brittle fracture and secondary microcracks (MAG 1.00 k×); (**C**) detail of the homogeneous micro-structure of the matrix without any porosity (MAG 1.00 k×).

**Figure 26:**
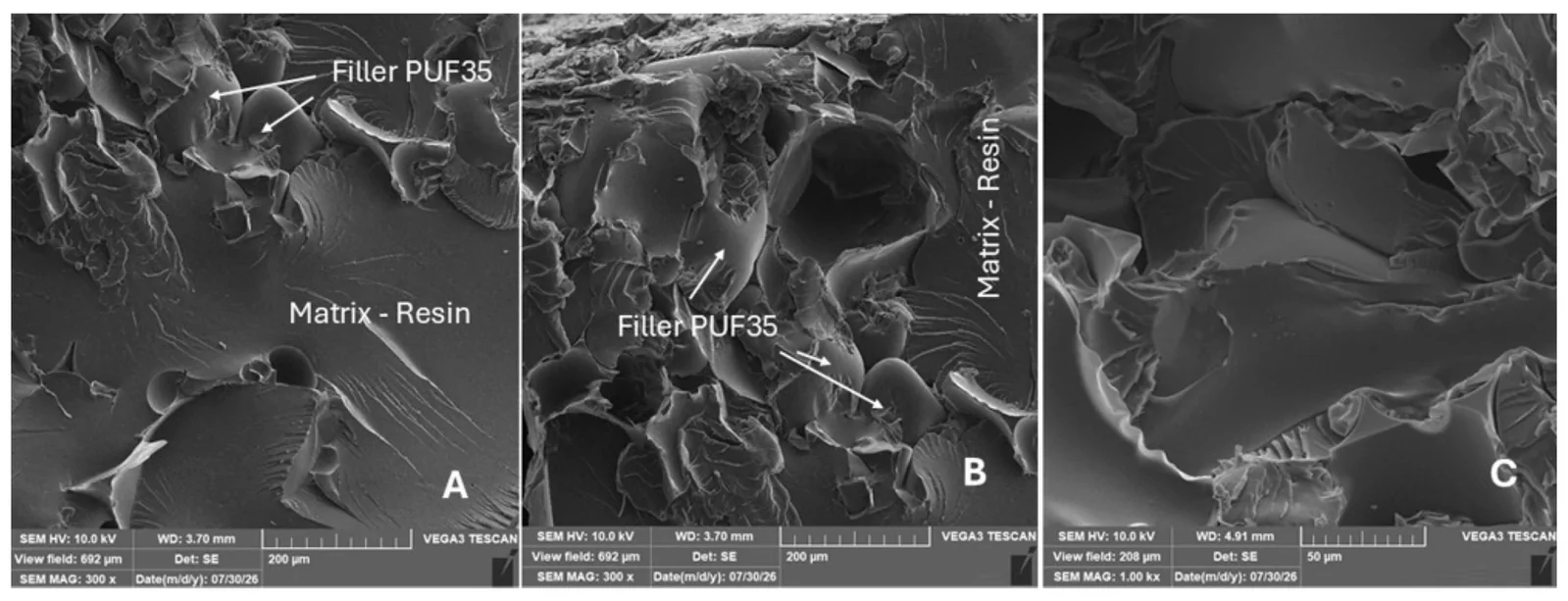
SEM micrographs of the fracture surface of a polymer matrix with PUF35 filler: (**A**) fracture surface oriented parallel to the direction of loading, showing the interphase interface of the composite system (MAG 300×); (**B**) a detail of the fracture surface in a plane perpendicular to the direction of loading, showing a clear crack deflection mechanism at the filler particles (MAG 300×); (**C**) a detailed view of the interphase region exhibiting high cohesion and the absence of defects at the matrix–filler inter-face (MAG 1.00 k×).

**Figure 27:**
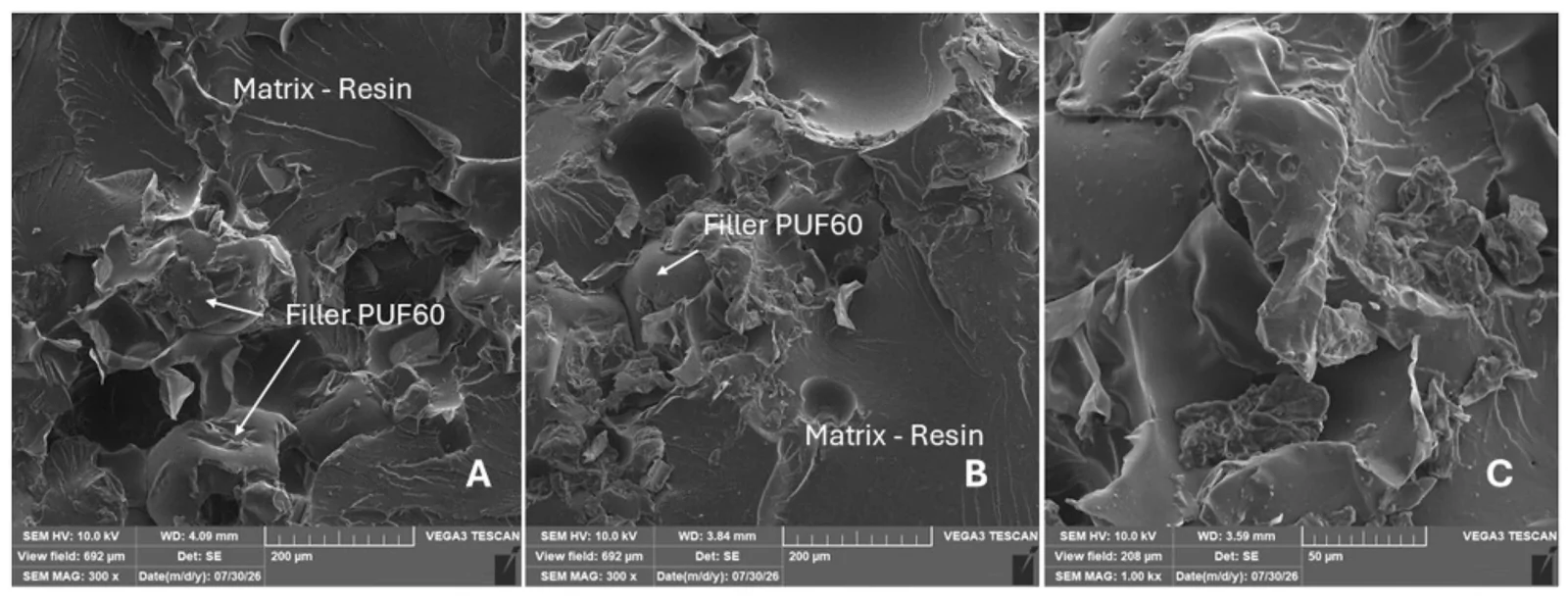
SEM micrographs of the fracture surface of a polymer matrix with PUF60 filler: (**A**) fracture surface oriented parallel to the direction of loading, showing the interphase boundary between the adhesive layer and the filler particles (MAG 300×); (**B**) a detail of the fracture surface oriented perpendicular to the direction of the applied load, showing the barrier function of the particles against crack propagation (MAG 300×); (**C**) a detailed view of the interphase region exhibiting high cohesion and the absence of defects at the matrix–filler interface (MAG 1.00 k×).

**Figure 28:**
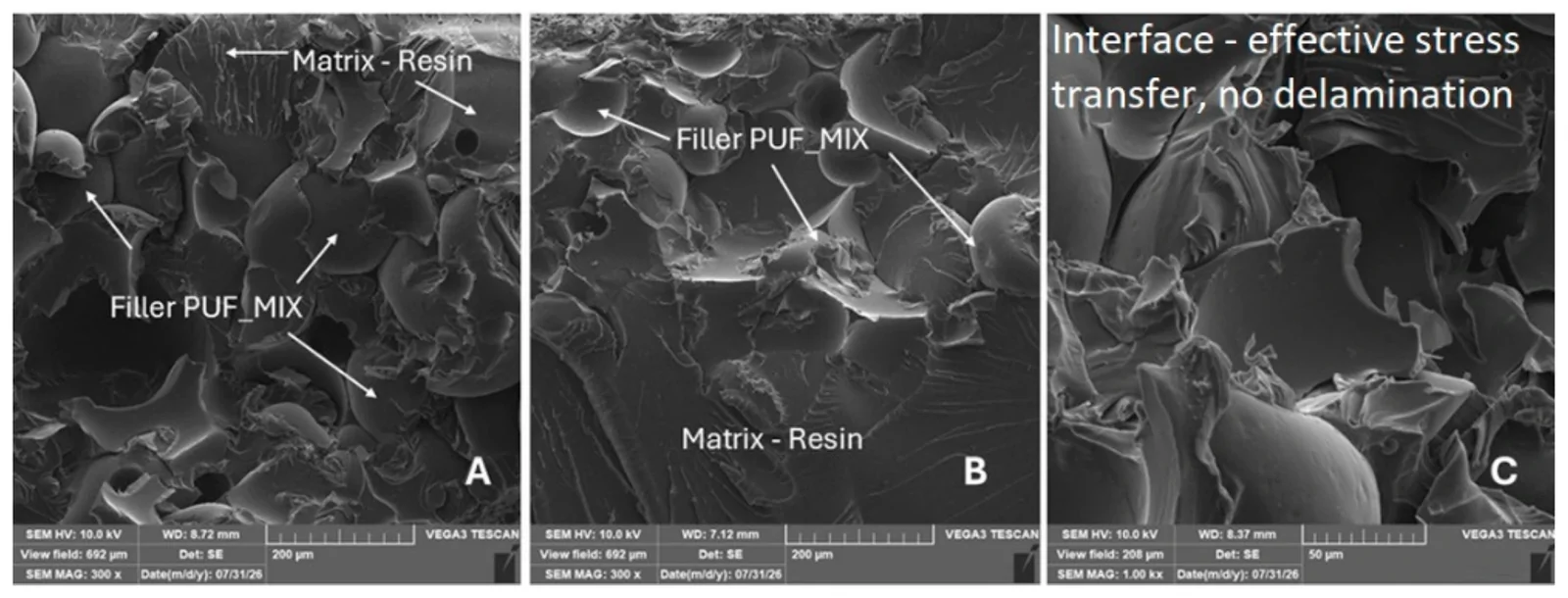
SEM micrographs of the fracture surface of a polymer matrix with PUF_MIX hybrid filler: (**A**) fracture surface oriented parallel to the direction of loading, showing the phase interface between the adhesive layer and the particles of the hybrid filler mixture (MAG 300×); (**B**) a detail of the fracture surface oriented perpendicular to the direction of the applied load, showing the barrier function of the mixture particles against crack propagation (MAG 300×); (**C**) a detailed view of the structural interface indicating good interaction and cohesion between the matrix and the hybrid filler (MAG 1.00 k×).

The SEM micrographs in Figure 25, Figure 26, Figure 27 and Figure 28 show fracture surfaces parallel (A) and perpendicular (B) to the loading direction, while higher-magnification images (C) show the matrix–filler interface and interfacial characteristics.

The reference specimen (Figure 25) shows a smooth fracture surface with characteristic river patterns (Figure 25A). The perpendicular surface (Figure 25B) exhibits brittle fracture and microcracks, while Figure 25C shows a homogeneous matrix without structural defects, micropores, or foreign inclusions.

SEM analysis of the PUF35 composite (Figure 26) shows a relatively homogeneous filler distribution and a distinct matrix–filler interface. Figure 26A presents the fracture surface parallel to the applied load, while Figure 26B shows the perpendicular plane, where increased surface irregularity and changes in the crack path are visible in the vicinity of the PUF particles. Higher magnification (Figure 26C) shows close matrix–filler contact without significant micro-delamination or critical interfacial defects.

The SEM micrographs of the PUF60 composite (Figure 27) show a relatively homogeneous filler distribution and a distinct matrix–filler interface. Figure 27A presents the fracture surface parallel to the applied load, while Figure 27B shows the perpendicular plane. Higher magnification (Figure 27C) shows close matrix–filler contact without significant micro-delamination or critical interfacial defects.

The SEM micrographs in Figure 28 document the fracture morphology of the composite system containing the PUF35/60 filler. Figure 28A shows the matrix–filler interface and a relatively homogeneous distribution of particles within the analysed adhesive region. Figure 28B reveals increased fracture-surface irregularity and local crack deflection associated with the presence of the filler. The detailed view in Figure 28C shows close matrix–filler contact without significant interfacial delamination or critical structural defects.

## 4. Discussion

### 4.1. Effect of Recycled PUF Morphology on Interfacial Interactions

As shown by the SEM analysis presented in Section 3.1, the more compact morphology of the PUF60 filler is likely related to the higher bulk density of the original foam. Its pronounced surface roughness may contribute to mechanical anchoring in the polymer matrix. The greater variability in particle size and shape observed for the PUF35/60 filler reflects the combination of polyurethane foams with different densities. The distribution of finer and coarser fractions may contribute to more efficient filling of the matrix. The observed particle morphology can also be related to the rigid closed-cell structure of the original PUF. Mechanical crushing disrupted the original cellular structure, resulting in irregular particles with rough surfaces, locally preserved cell walls, and cavities.

### 4.2. Effect of Recycled PUF on the Mechanical Behaviour of Rectangular-Core Composites

As shown by the tensile strength and modulus of elasticity results for composites with the rectangular honeycomb core presented in Section 3.2, the mechanical response depended on both the type and content of recycled PUF. The favourable performance of PUF35/60, particularly compared with the single-density PUF35 and PUF60 fillers, may be associated with a more homogeneous particle distribution and more efficient load transfer between the epoxy matrix, recycled PUF particles, and the 3D-printed rectangular core. In contrast, higher contents of single-density PUF may promote local microstructural inhomogeneities and stress concentrations, thereby reducing the efficiency of load transfer. Similar effects of microstructural homogeneity, particle distribution, and stress concentrations on load transfer and mechanical performance have been reported by Hedjazi et al. [59] and Song et al. [60]. The importance of filler topology and geometry for stress distribution and the mechanical behaviour of FDM structures has also been demonstrated by Dezaki et al. [41], Cabreira et al. [61], and Cojocaru et al. [62].

The modulus of elasticity results presented in Section 3.2 further support the influence of PUF type and content on the mechanical behaviour of the composites. The generally higher modulus values achieved by PUF35/60 compared with PUF35 and, at some concentrations, PUF60 may similarly be associated with a more homogeneous filler distribution and improved load transfer between the matrix, PUF particles, and rectangular core [59,60]. The rectangular core geometry may additionally contribute to the stiffness of the composite through a more uniform stress distribution, consistent with previous studies on FDM structures [41,61].

The decrease in modulus of elasticity observed with increasing PUF35 content may be associated with disruption of the continuity of the epoxy matrix by the lower-stiffness PUF particles. In contrast, PUF60 maintained relatively higher modulus values at lower filler concentrations, followed by a marked decrease at higher concentrations. This behaviour may result from local inhomogeneities or particle agglomeration, which can reduce load-transfer efficiency [45]. The importance of microstructural homogeneity and uniform stress distribution for mechanical performance is also supported by Hedjazi et al. [59].

### 4.3. Effect of Recycled PUF on the Mechanical Behaviour of Hexagonal-Core Composites

As shown by the tensile strength results for composites with the hexagonal honeycomb core presented in Section 3.3, lower recycled PUF contents did not substantially reduce tensile strength, whereas higher filler contents resulted in a gradual decrease. A similar behaviour was observed for composites with the rectangular core (Section 3.2). PUF35/60 generally maintained higher tensile strength than PUF35 and PUF60 at higher filler concentrations, which may be associated with a more homogeneous particle distribution and more efficient load transfer within the composite. The importance of homogeneous particle distribution and microstructure for reducing local stress concentrations and maintaining mechanical performance has also been reported by Song et al. [60] and Hedjazi et al. [59].

The hexagonal core can promote relatively uniform stress distribution and efficient load transfer while providing high stiffness at low weight [40,41,61]. Previous studies have also highlighted the importance of core geometry and core–filler bonding for the mechanical behaviour of honeycomb structures [47,63]. However, since the hexagonal core geometry was identical for all variants investigated in Section 3.2, the differences in tensile strength within this group can primarily be associated with changes introduced by recycled PUF into the epoxy matrix rather than with changes in core geometry.

The modulus of elasticity results presented in Section 3.3 showed a similar dependence on recycled PUF type and content. The generally higher modulus values obtained for PUF35/60 may be associated with a more homogeneous filler distribution and improved load transfer [59]. Uniform particle dispersion can reduce local stress concentrations and thereby contribute to improved mechanical performance [45]. This interpretation is also consistent with the SEM observations presented in Section 3.4, where the PUF35/60 filler exhibited a combination of finer and coarser particles without significant agglomeration.

The hexagonal core may additionally contribute to uniform load distribution and high stiffness [41,61], while cell topology can significantly affect the mechanical response of such structures [40]. The performance of filled honeycomb structures also depends on core geometry and core–filler bonding [60,63]. Since the same hexagonal core geometry was used for all variants, the differences observed among the investigated samples were primarily associated with the modification of the epoxy matrix by recycled PUF.

Overall, the results presented in Section 3.2 and Section 3.3 demonstrate that the mechanical response of the epoxy-based composite system depends on both the type and concentration of the particulate recycled PUF filler. An appropriately selected filler type and concentration can maintain or improve certain mechanical properties, whereas higher filler concentrations can lead to their deterioration. This behaviour is consistent with previous studies reporting that the mechanical performance of epoxy/polymer systems strongly depends on the type and concentration of the incorporated filler [64,65].

### 4.4. Effect of Microstructural Characteristics on the Mechanical Behaviour of Hybrid Composites

The microstructural observations presented in Section 3.4 provide further insight into the behaviour of the investigated composites. The absence of significant particle pull-out or interfacial separation for PUF35 and PUF60 indicates good adhesion between the recycled PUF particles and the epoxy matrix. Similarly, the uniform particle dispersion and continuous PLA–composite interface observed for PUF35/60 indicate favourable interaction between the individual components of the composite.

Compared with the reference sample, the rougher fracture surfaces and more complex crack paths observed in the PUF-containing composites can be associated with the presence and irregular morphology of the recycled PUF particles. Although local voids and irregularities were observed, mainly in connection with the porous morphology of PUF, no significant porosity disrupting the composite layer was identified.

The close contact between the epoxy and PLA surfaces observed in Section 3.4, together with the penetration of epoxy into local FDM surface irregularities, may promote mechanical anchoring at the PLA–epoxy interface. Although local printing defects were observed, particularly for PUF60 and PUF35/60, the absence of associated crack initiation or interfacial separation indicates that these defects did not act as preferential failure sites under the investigated conditions.

These microstructural observations are consistent with the mechanical results presented in Section 3.2 and Section 3.3. Among the investigated filler types, PUF35/60 showed the most favourable overall mechanical response, providing the highest tensile strength for the rectangular-core composites and the highest modulus of elasticity for both core geometries. Its relatively uniform particle dispersion and continuous interface observed by SEM may contribute to this mechanical behaviour.

The results also show that favourable mechanical performance was achieved without additional filler pretreatment, although chemical or physical surface modifications are commonly used to improve matrix compatibility and interfacial adhesion [66,67].

### 4.5. Effect of Recycled PUF on the Mechanical Behaviour of Overlapped Joints

The adhesive bond strength results presented in Section 3.5 show that the effect of recycled PUF on the lap joints depended on both filler content and density. While the addition of 1 wt% PUF35 maintained the adhesive bond strength at a level comparable to the unfilled reference joint, higher PUF35 contents were associated with a gradual decrease in bond strength. In contrast, no clear concentration-dependent trend was observed for PUF60 and PUF35/60. A similar absence of a clear relationship between recycled PUF content and bond strength was reported by Hýsek et al. [4], although a one-component polyurethane adhesive was used in their study rather than the epoxy resin applied in the present work.

The observed reduction in adhesive bond strength may be related to the properties and distribution of recycled PUF particles within the adhesive layer. Bond strength is influenced by both adhesive composition and adhesive–adherend interactions [68]. Increasing the particulate filler content may disrupt the continuity of the epoxy matrix, promote local inhomogeneities, and consequently reduce load-transfer efficiency between the adhesive and adherends.

The modulus of elasticity results for the laminated joints presented in Section 3.5 demonstrate different responses depending on the type and content of recycled PUF. PUF35 initially reduced the modulus of elasticity, after which the values gradually returned to a level comparable to the reference joint. In contrast, PUF60 resulted in an increase in the modulus of elasticity at higher filler contents, while PUF35/60 exhibited a less pronounced and non-linear response. These differences may reflect changes in the stiffness of the modified epoxy layer, as denser PUF particles at higher contents may reduce matrix deformability. However, increased stiffness does not necessarily result in improved adhesive bond strength and may promote local stress concentrations and failure initiation.

Overall, the differences between the mechanical behaviour of the hybrid structures presented in Section 3.2 and Section 3.3 and the bonded joints presented in Section 3.5 can be attributed to their different load-transfer mechanisms. In the 3D-printed hybrid structures, the PLA core contributes to structural stiffness and load transfer and can therefore partially compensate for changes in the mechanical properties of the PUF-modified epoxy matrix. In contrast, in the bonded joints, the PUF-modified epoxy directly forms the adhesive layer responsible for load transfer between the adherends. At higher filler contents, disruption of the epoxy matrix continuity and local inhomogeneities may therefore have a more direct negative effect on the adhesive bond strength.

### 4.6. Fracture Behaviour and Interfacial Characteristics of Overlapped Joints

The fracture-surface characteristics of the bonded joints presented in Section 3.6 provide further information on the failure mechanisms and interfacial behaviour of the investigated adhesive systems. The mixed adhesive–cohesive fracture (ACF) observed in the bonded joints indicates simultaneous crack propagation within the adhesive layer and at the adhesive–adherend interface. Such a mixed failure mode is common in bonded metal joints and generally indicates a high level of adhesion at the interface [58,69]. The absence of substantial changes in the overall fracture mode after incorporation of recycled PUF suggests that, at the investigated concentrations, the filler did not fundamentally alter the mechanism of fracture initiation and propagation. Similar observations have been reported in previous studies of bonded joints containing other filler types [58].

The SEM observations presented in Section 3.6 also indicate favourable interaction between the recycled PUF particles and the epoxy matrix. The relatively homogeneous filler distribution and absence of significant micro-delamination or critical interfacial defects observed for PUF35, PUF60, and PUF35/60 suggest favourable matrix–filler adhesion. The increased fracture-surface irregularity and local crack deflection observed in the presence of PUF particles may be associated with changes in crack propagation through the modified epoxy layer. The close matrix–filler contact observed at higher magnification may also contribute to stress transfer between the epoxy matrix and recycled PUF particles.

However, the favourable local interfacial characteristics observed by SEM did not necessarily result in improved macroscopic adhesive bond strength. As shown by the mechanical results presented in Section 3.5, 5 wt% PUF35/60 resulted in an approximately 6.9% decrease in adhesive bond strength compared with the unfilled reference joint. This indicates that local interfacial quality alone does not govern the mechanical performance of the thin adhesive layer. Other filler-related effects, including disruption of epoxy matrix continuity and local heterogeneities, may also contribute to the reduction in joint strength.

### 4.7. Dispersion and Interfacial Bonding Mechanism of Recycled PUF in the Epoxy Matrix

The SEM observations of filler morphology (Section 3.1) and fracture surfaces (Section 3.4 and Section 3.6) provide insight into the dispersion and interfacial interaction of recycled PUF within the epoxy matrix. The irregular particle morphology resulting from mechanical crushing, including rough surfaces, sharp edges, cavities, and locally preserved fragments of the original cellular structure, increases the available interfacial area and may provide favourable conditions for close matrix–filler contact and mechanical interlocking after curing.

Within the SEM regions examined, the relatively homogeneous distribution of PUF particles and the absence of pronounced interfacial gaps, extensive particle pull-out, or significant matrix–filler delamination indicate favourable interfacial interaction. Together with the increased fracture-surface irregularity and local crack deflection observed in the PUF-containing specimens, these findings support a predominantly morphological and mechanical contribution to the PUF–epoxy interfacial interaction.

These microstructural observations can also be related to the mechanical behaviour discussed in Section 4.2, Section 4.3, Section 4.4 and Section 4.5. PUF35/60 showed the most favourable response in the hybrid structures, whereas satisfactory local matrix–filler cohesion in the bonded joints did not necessarily result in improved adhesive bond strength. This indicates that local interfacial quality alone does not govern the macroscopic performance of the composite system. Particularly in thin adhesive layers, other filler-related effects, including changes in mixture viscosity, matrix continuity, local heterogeneity, and stress distribution, may become increasingly important with increasing filler content.

Possible physicochemical interactions between polyurethane and the epoxy matrix, including hydrogen bonding or other polar interactions, may complement the mechanical interlocking mechanism. However, because the detailed chemical composition of the recycled PUF was unavailable and no spectroscopic analysis of the interface was performed, such interactions cannot be confirmed in the present study.

## 5. Conclusions

The research evaluated the environmentally friendly use of mechanically recycled PUF as a particulate filler in hybrid composite systems with 3D-printed PLA honeycomb cores and bonded lap joints. The main conclusions are as follows:

- Filler morphology and dispersion: SEM analysis showed relatively homogeneous dispersio3.5n of PUF35, PUF60, and PUF35/60 without significant agglomeration. The irregular and rough particle morphology supported mechanical interlocking and good adhesion within the epoxy matrix.
- Hybrid composite structures: The mechanical performance was influenced by filler content and core geometry. PUF35/60 provided the most favourable results. At 4 wt% PUF35/60, the modulus of elasticity increased by approximately 12.8% for the rectangular core and 13.9% for the hexagonal honeycomb core compared with the unfilled reference composites. At 3 wt% PUF35/60, the tensile strength of the rectangular-core composite increased by approximately 4.4%, while the tensile strength of the hexagonal-core composite remained at the reference level. Statistical analysis confirmed significant relationships between PUF content and mechanical properties for most PUF35 and PUF60 variants, while PUF35/60 generally exhibited weaker relationships.
- Bonded joints: Recycled PUF did not significantly improve adhesive bond strength. For 1 wt% PUF35, the bond strength remained at the reference level, while 5 wt% PUF35 resulted in an approximately 14.5% decrease. For 5 wt% PUF60, the decrease was also approximately 10.7%, while 5 wt% PUF35/60 resulted in an approximately 6.9% decrease. Statistical analysis confirmed significant relationships for PUF35 and PUF60 in shear strength, while a significant increase in the modulus of elasticity was observed only for PUF60. These results indicate that the benefits of recycled PUF are more pronounced in hybrid composite structures than in thin adhesive layers.

Overall, mechanically recycled PUF, particularly PUF35/60, shows potential as a filler for hybrid composite structures while providing a possible route for the utilisation of polyurethane waste. Future research should focus on optimising PUF dispersion and PUF–epoxy interfacial bonding, including surface treatments, as well as investigating long-term durability and environmental resistance to further improve the practical applicability of these sustainable hybrid composites.

## Data Availability

The original contributions presented in this study are included in the article. Further inquiries can be directed to the corresponding authors.

## References

[1] Rossignolo G., Malucelli G., Lorenzetti A. (2024). Recycling of polyurethanes: Where we are and where we are going. Green Chem..

[2] Gama N., Godinho B., Marques G., Silva R., Barros-Timmons A., Ferreira A.J. (2020). Recycling of polyurethane scraps via acidolysis. Chem. Eng. J..

[3] Kemona A., Piotrowska M. (2020). Polyurethane Recycling and Disposal: Methods and Prospects. Polymers.

[4] Hýsek Š., Sedivka P., Böhm M., Schönfelder O., Beran R. (2018). Influence of Using Recy-cled Polyurethane Particles as a Filler on Properties of Polyurethane Adhesives for Gluing of Wood. BioResources.

[5] Beran R., Zarybnicka L., Machova D. (2020). Recycling of rigid polyurethane foam: Micromilled powder used as active filler in polyurethane adhesives. J. Appl. Polym. Sci..

[6] Xi W., Qian L., Li L. (2019). Flame Retardant Behavior of Ternary Synergistic Systems in Rigid Polyurethane Foams. Polymers.

[7] Bhatti H., Ahmad I. (2007). Methods for polyurethane and polyurethane composites, recycling and recovery: A review. React. Funct. Polym..

[8] Gama N., Godinho B., Madureira P., Marques G., Barros-Timmons A., Ferreira A. (2024). Polyurethane Recycling Through Acidolysis: Current Status and Prospects for the Future. J. Polym. Environ..

[9] Grdadolnik M., Drincic A., Oreski A., Onder O.C., Utrosa P., Pahovnik D., Zagar E. (2022). Insight into Chemical Recycling of Flexible Polyurethane Foams by Acidolysis. ACS Sustain. Chem. Eng..

[10] Banik J., Chakraborty D., Rizwan M., Shaik A.H., Chandan M.R. (2023). Review on disposal, recycling and management of waste polyurethane foams: A way ahead. Waste Manag. Res..

[11] Zarezadeh E., Tangestani M., Jafari A.J. (2024). A systematic review of methodologies and solutions for recycling polyurethane foams to safeguard the environment. Heliyon.

[12] Selvaraj V.K., Subramanian J., Mouleswaran S., Keerthan T.K., Muneeswaran T., Nath A.K., Raju M.P. (2025). Sustainable development of bioepoxy composites reinforced with recycled rigid polyurethane foam for mechanical, thermal, acoustic, and electromag-netic applications in a circular economy approach. Sci. Rep..

[13] Schyns Z.O.G., Shaver M.P. (2021). Mechanical Recycling of Packaging Plastics: A Review. Macromol. Rapid Commun..

[14] Deng Y., Dewil R., Appels L., Ansart R., Baeyens J., Kang Q. (2021). Reviewing the thermo-chemical recycling of waste polyurethane foam. J. Environ. Manag..

[15] Yang W., Dong Q., Liu S., Xie H., Liu L., Li J. (2012). Recycling and Disposal Methods for Polyurethane Foam Wastes. Procedia Environ. Sci..

[16] Günther M., Lorenzetti A., Schartel B. (2018). Fire Phenomena of Rigid Polyurethane Foams. Polymers.

[17] Guo L., Wang W., Guo X., Hao K., Liu H., Xu Y., Liu G., Guo S., Bai L., Ren D. (2022). Recycling of Flexible Polyurethane Foams by Regrinding Scraps into Powder to Replace Polyol for Re-Foaming. Materials.

[18] Simón D., Rodríguez J.F., Carmona M., Serrano A., Borreguero A.M. (2018). Glycolysis of advanced polyurethanes composites containing thermoregulating microcapsules. Chem. Eng. J..

[19] Simón D., Borreguero A.M., de Lucas A., Rodríguez J.F. (2014). Glycolysis of flexible polyurethane wastes containing polymeric polyols. Polym. Degrad. Stab..

[20] Calvo-Correas T., Ugarte L., Trzebiatowska P.J., Sanzberro R., Datta J., Corcuera M.Á., Eceiza A. (2017). Thermoplastic polyurethanes with glycolysate intermediates from polyurethane waste recycling. Polym. Degrad. Stab..

[21] Simón D., Borreguero A.M., de Lucas A., Rodríguez J.F. (2018). Recycling of polyure-thanes from laboratory to industry, a journey towards the sustainability. Waste Manag..

[22] Gama N.V., Ferreira A., Barros-Timmons A. (2018). Polyurethane Foams: Past, Present, and Future. Materials.

[23] Eling B., Tomović Ž., Schädler V. (2020). Current and Future Trends in Polyurethanes: An Industrial Perspective. Macromol. Chem. Phys..

[24] Salino R.E., Catai R.E. (2023). A study of polyurethane waste composite (PUR) and recycled plasterboard sheet cores with polyurethane foam for acoustic absorption. Constr. Build. Mater..

[25] Mark F.E., Kamprath A. Recycling & Recovery Options for PU Seating Material: A Joint Study of ISOPA/Euro-Moulders. Proceedings of the 2000 SAE Total Life Cycle Conference and Exposition.

[26] Chen S., Wang F., Peng Y., Chen T., Wu Q., Sun P. (2015). A Single Molecular Diels–Alder Crosslinker for Achieving Recyclable Cross-Linked Polymers. Macromol. Rapid Commun..

[27] Wieczorek K., Bukowski P., Stawiński K., Ryłko I. (2024). Recycling of Polyurethane Foams via Glycolysis: A Review. Materials.

[28] Hosgor E., Martinho R.P., Hoogland J.S., Jia Y., Morales Gomez A., Verboom W., Lange J.P., Huskens J. (2025). Polyurethane depolymerization by dialkyl carbonates: Toward sustainable chemical recycling. Green Chem..

[29] Delavarde A., Savin G., Derkenne P., Boursier M., Morales-Cerrada R., Nottelet B., Pinaud J., Caillol S. (2024). Sustainable polyurethanes: Toward new cutting-edge opportunities. Prog. Polym. Sci..

[30] Mansouri H.R., Pizzi A. (2007). Recycled micronized polyurethane powders as active extenders of UF and PF wood panel adhesives. Holz Als Roh Werkst..

[31] Qu C.B., Huang G.W., Li N., Li M., Ma J.-L., Liu Y., Xiao H.M. (2021). Improving cryogenic mechanical properties of carbon fiber reinforced composites based on epoxy resin toughened by hydroxyl-terminated polyurethane. Compos. Part B Eng..

[32] Seo J., Lee D., Ji H., Yang S.B., Baek Y., Shin P.S., Kim M.T., Kwon D.J. (2026). Enhanced interlaminar adhesion and stab resistance in recycled composites using waste prepreg and milled waste polyurethane foam. Compos. Part B Eng..

[33] Liang F., Chen Q., Shi Z., Dan R., Liang H., Feng X., Tang C., Yu X., Shang X., Zhang D. (2025). Multifunctional composites made from waste polyester cotton fabric and waste rigid polyurethane foam for potential building wall material applications. Energy Build..

[34] Xia P., Liu Q., Fu H., Yu Y., Wang L., Wang Q., Yu X., Zhao F. (2023). Mechanical properties and energy absorption of 3D printed double-layered helix honeycomb under in-plane compression. Compos. Struct..

[35] Townsend S., Adams R., Robinson M., Hanna B., Theobald P. (2020). 3D printed origami honeycombs with tailored out-of-plane energy absorption behavior. Mater. Des..

[36] Kendall P., Sun M., Wowk D., Mechefske C., Kim I.Y. (2020). Experimental Investigation of Adhesive Fillet Size on Barely Visible Impact Damage in Metallic Honeycomb Sandwich Panels. Compos. Part B Eng..

[37] Jiang H., Ji Y., Hu Y., Hu X., Ren Y. (2022). Interfacial Design and Flexural Property of CFRP/Aluminum-Honeycomb Sandwich with Aramid-Pulp Micro/Nano-Fiber Inter-lays. Compos. Struct..

[38] Pereira A.B., Fernandes F.A.O. (2019). Sandwich Panels Bond with Advanced Adhesive Films. J. Compos. Sci..

[39] Zebrine D., Anders M., Centea T., Nutt S. (2020). Path-Dependent Bond-Line Evolution in Equilibrated Core Honeycomb Sandwich Structures. Adv. Manuf. Polym. Compos. Sci..

[40] Mahović Poljaček S., Donevski D., Tomašegović T., Vrabič Brodnjak U., Leskovšek M. (2025). Mechanical Properties of 3D-Printed PLA Structures Observed in Framework of Different Rotational Symmetry Orders in Infill Patterns. Symmetry.

[41] Lalegani Dezaki M., Mohd Ariffin M.K.A. (2020). The Effects of Combined Infill Patterns on Mechanical Properties in FDM Process. Polymers.

[42] Ćwikła G., Grabowik C., Kalinowski K., Paprocka I., Ociepka P. (2017). The influence of printing parameters on selected mechanical properties of FDM/FFF 3D-printed parts. IOP Conf. Ser. Mater. Sci. Eng..

[43] Galeja M., Hejna A., Kosmela P., Kulawik A. (2020). Static and Dynamic Mechanical Properties of 3D Printed ABS as a Function of Raster Angle. Materials.

[44] Wang S., Pei W., Jin S., Yu H. (2024). Numerical and theoretical analysis of the out-of-plane crushing behavior of a sinusoidal-shaped honeycomb structure with tunable mechanical properties. Structures.

[45] Song X., Hong S., Wang J., Zhu X., Guo S., Fu Y., Yang Y., Yang M., He W., Tang Y. (2024). Mechanical Properties of a Honeycomb Structure Dispersed with 3D-Printed Fe3O4 Nanomaterials. ACS Omega.

[46] Jin T., Zhou Z., Wang Z., Wu G., Shu X. (2015). Experimental study on the effects of specimen in-plane size on the mechanical behavior of aluminum hexagonal honeycombs. Mater. Sci. Eng. A.

[47] Song S., Xiong C., Tao F., Yin J., Zhang Y., Zhang S. (2022). Size effect of composite Kagome honeycomb sandwich structure rein-forced with PMI foams under quasi-static bending: Experiment tests and numerical analysis. Compos. Struct..

[48] Yang X., Ma J., Sun Y., Yang J. (2018). Ripplecomb: A novel triangular tube reinforced corrugated honeycomb for energy absorption. Compos. Struct..

[49] Qi J., Li C., Tie Y., Zheng Y., Duan Y. (2021). Energy absorption characteristics of origami-inspired honeycomb sandwich structures under low-velocity impact loading. Mater. Des..

[50] Du Z.H., Ma S.B., Han G.Q., Wei X., Han J.C., Zhang K.F. (2021). The parameter optimization and mechanical property of the honeycomb structure for Ti2AlNb based alloy. J. Manuf. Process..

[51] Müller M., Jirků P., Šleger V., Mishra R.K., Hromasová M., Novotný J. (2022). Effect of Infill Density in FDM 3D Printing on Low-Cycle Stress of Bamboo-Filled PLA-Based Material. Polymers.

[52] Lalegani Dezaki M., Ariffin M.K., Serjouei A., Zolfagharian A., Hatami S., Bodaghi M. (2021). Influence of Infill Patterns Generated by CAD and FDM 3D Printer on Surface Roughness and Tensile Strength Properties. Appl. Sci..

[53] Jirků P., Muller M., Mishra R.K., Svobodová J. (2025). Effect of Recycling and UV Ageing on the Properties of PLA-Based Materials Used in Additive Manufacturing. Polymers.

[54] (2020). Plasty—Stanovení Tahovych Vlastností—část 1: Obecné Principy.

[55] (2009). Adhesives—Determination of Tensile Lap-Shear Strength of Bonded Assemblies.

[56] Rudawska A. (2019). Surface Treatment in Bonding Technology.

[57] van Dam J.P.B., Abrahami S.T., Yilmaz A., Gonzalez-Garcia Y., Terryn H., Mol J.M. (2020). Effect of surface roughness and chemistry on the adhesion and durability of a steel-epoxy adhesive inteface. Int. J. Adhes. Adhes..

[58] Müller M., Svobodová J., Hrabě P., Mishra R.K., Valášek P., Rudawská A. (2026). Research on an innovative nettle-based filler in the field of hybrid adhesive joints. J. Adhes. Sci. Technol..

[59] Hedjazi L., Belhabib S., D’Orlando A., Guessasma S. (2023). Breaking Material Symmetry to Control Mechanical Performance in 3D Printed Objects. Symmetry.

[60] Song S., Xiong C., Yin J., Zhang S. (2024). Mechanical property of all-composite diamond honeycomb sandwich structure based on interlocking technology: Experimental tests and numerical analysis. Mech. Adv. Mater. Struct..

[61] Cabreira V., Santana R. (2020). Effect of infill pattern in Fused Filament Fabrication (FFF) 3D Printing on materials performance. Matér. Rio Jan..

[62] Cojocaru V., Frunzaverde D., Miclosina C.-O. (2023). On the Behavior of Honeycomb, Grid and Triangular PLA Structures under Symmetric and Asymmetric Bending. Micromachines.

[63] Xu H.H., Luo H.C., Zhang X.G., Jiang W., Teng X.C., Chen W.Q., Yang J., Xie Y.M., Ren X. (2023). Mechanical properties of aluminum foam filled reentrant honeycomb with uniform and gradient designs. Int. J. Mech. Sci..

[64] Abbasi Z., Jazani O.M., Sohrabian M. (2021). Designing of high-performance epoxy adhesive with recycled polymers and silica nano particles (SNPs) in epoxy/carbon fiber composite-steel bonded joints: Mechanical properties, thermal stability and toughen-ing mechanisms. J. Taiwan Inst. Chem. Eng..

[65] Ogrodniczek J., Rudawska A., Müller M. (2025). Comparative analysis of compressive strength of epoxy adhesive compounds containing fillers. J. Adhes..

[66] Fan T., Hu R., Zhao Z., Liu Y., Lu M. (2017). Surface micro-dissolve method of imparting self-cleaning property to cotton fabrics in NaOH/urea aqueous solution. Appl. Surf. Sci..

[67] Müller M., Valášek P., Rudawska A. (2017). Mechanical properties of adhesive bonds reinforced with biological fabric. J. Adhes. Sci. Technol..

[68] Banea M.D., da Silva L.F.M. (2009). Adhesively bonded joints in composite materials: An overview. Proc. Inst. Mech. Eng. Part J. Mater. Des. Appl..

[69] Kinloch A.J. (1979). Interfacial Fracture Mechanical Aspects of Adhesive Bonded Joints—A Review. J. Adhes..

